# Supplementary material for: A pilot multicentre cluster randomised trial to compare the effect of trauma life support training programmes on patient and provider outcomes
Source: BMJ Open. 2022 Apr 15;12(4):e057504. doi: 10.1136/bmjopen-2021-057504 (PMC9016405; doi:10.1136/bmjopen-2021-057504)
Supplement: Supplementary data [file bmjopen-2021-057504supp001.pdf]

Supplemental Material 1

1.1. Study Enrolment: Patient Information Sheet

Patient Information Sheet

You are being invited to participate in a research study. Before your data can be included in this data bank the purpose of the data collection must be explained to you, and you must be given the chance to ask questions. Please read carefully the information provided here. If you agree to participate, please sign the informed consent form. You will be given a copy of this document to take home with you.

Protocol Title: Trauma Training Effectiveness Research Network

Principal Investigator: Name

PURPOSE OF THE RESEARCH STUDY

We are currently conducting research in this hospital to study the feasibility of assessing the effect of trauma life support training programs on care and outcome of patients with injury. We ask you to participate in this study because you presented to this hospital after having an injury.

STUDY PROCEDURES AND VISIT SCHEDULE

If you agree to participate, we will:

- Store health data registered in your hospital records and vital signs recordings from the emergency department
- Contact you in person or by telephone for follow ups to obtain information about your health status at the following times:
    - On arrival at the emergency department and wards till you are discharged
    - On hospital discharge
    - 24 hours after arrival to the hospital
    - 30 days after arrival to the hospital

When you arrived at the hospital, we recorded some basic parameters such as your age, gender, and how you were injured. We also recorded health data such as blood pressure, heart rate, oxygen levels, respiratory rate, surgical care and treatment provided. During your stay, we will record periodically health data, the investigations and treatment that you have undergone. During the follow-up calls, we may ask you details about your health and general information on returning back to your normal life and experience of the injury in your life. If you want complete information regarding all the parameters that were recorded, please do not hesitate to ask, and we will be happy to inform you.

Should you wish that your data is deleted from the study, you may please tell us now or contact us using the contact information provided below. The results of the study may be used for research that can be published as scientific articles; however, it will not be possible to identify you by reading any article that may result from this data bank. Further, data from this project will be combined with data from other hospitals that use the same system and shared for other researchers and individuals to use, but it will not be possible to identify you using that data. Research on the data without identifiers may seek to answer other questions than those stated above.

WITHDRAWAL FROM STUDY

Participation in this study is completely voluntary. Even if you agree to participate now you are free to withdraw at any time without giving any reason for doing so. Withdrawing will not affect your ordinary treatment or the care given to you. To withdraw contact any of the contact persons using the contact information provided. Note that we can only delete data from the data collected in this hospital. We cannot delete data once it has been de-identified because we will not be able to tell from whom the data came.

POSSIBLE RISKS, DISCOMFORTS AND INCONVENIENCES

We have not been able to identify any major risks associated with participating in this study. Even if you agree to participate now you are free to withdraw at any time without giving any reason for doing so. You are free to withdraw at that point, or at any time using the contact information provided below.

POTENTIAL BENEFITS

This research may help to improve the care of injured patients. Although this study will not affect the care you are given in this hospital at this time, your participation will contribute to medical knowledge, and may improve care for you if you are injured again in the future care for others that are injured.

SUBJECT'S RIGHTS

Your participation in this study is entirely voluntary. Your questions will be answered clearly and to your satisfaction. In the event of any new information becoming available that may be relevant to your willingness to continue in this study, you will be informed in a timely manner by the Principal Investigator or his/her representative.

CONFIDENTIALITY OF STUDY AND MEDICAL RECORDS

The results of this research may be published as a scientific article; however, it will not be possible to identify you by reading any article that may result from this work. Further, data from this project will be combined with data from other hospitals that use the same system and shared for other researchers and individuals to use, but it will not be possible to identify you using that data.

Also, Regulatory Agencies, Institutional Review Board and Ministry of Health will be granted direct access to your original medical records to check study procedures and data, without

making any of your information public. By signing the Informed Consent Form attached, you are authorizing such access to your study and medical records.

COSTS OF PARTICIPATION

No charge will be levied on you if you take part in this study. You will not receive any compensation for participating in this study.

RESEARCH RELATED INJURY AND COMPENSATION

Due to the observational nature of this project, it is unlikely to cause any research related injury. The hospital will provide medical care for any problems that may arise during this study.

WHOM TO CONTACT IF YOU HAVE QUESTIONS

If you have questions regarding this study and your rights, or in the case of any injuries sustained during this project, you may contact the Principal Investigator:

Name
Designation, Department
Phone Number
Email

1.2 Study Enrolment: Patient Consent Form

Consent Form

<u>Protocol Title:</u> Trauma Training Effectiveness Research Network

Patient Details

Name: NRIC/PNR/SSN No.:

Address:

Date of birth _______________ Phone No:

dd/mm/yyyy

Phone number(s) of your relatives or friends that you agree we may contact, in case you do not answer your phone:

Part I – to be filled by the patient

I, ______________________________________ (Pt ID No._________________) **agree / do not agree** to participate in the project as described and, on the terms detailed in the Patient Information Sheet. The nature of my participation in the proposed project has been explained to me in _______________ by Dr/Mr/Ms ______________________. I have fully discussed and understood the purpose and procedures of this project. I have been given the Patient Information Sheet and the opportunity to ask questions about this project and have received satisfactory answers and information. I understand that my participation is voluntary and that I am free to withdraw at any time, without giving any reasons and without my medical care being affected. I also give permission for information in my medical records to be used for this project. In any event of publication and sharing of the data with other researchers and individuals, I understand that this information will not bear my name or other identifiers and that due care will be taken to preserve the confidentiality of this information.

__________________________________ _________________________

(Signature/Thumbprint (Right / Left) of Subject) (Date of signing)

Part II – to be filled by parent / legal guardian, where applicable

I, __________________________ hereby give consent for the above patient to participate in the proposed project. The nature, risks and benefits of the study have been explained clearly to me and I fully understand them.

____ _________________________

(Signature/Thumbprint (Right / Left) (Date of signing)

of parent /legal guardian]

Part III – to be filled witness, where applicable

An impartial witness should be present during the entire informed consent discussion if a subject or the subject's legally acceptable representative is unable to read. After the written informed consent form and any written information to be provided to subjects, is read and explained to the subject or the subject's legally acceptable representative, and after the subject or the subject's legally representative has orally consented to the subject's participation in the study and, if capable of doing so, has signed and personally dated the consent form, the witness should sign and personally date the consent form.

__________________________ __________________________

(Name of witness) (Designation of witness)

__________________________ __________________________

(Signature of witness) (Date of signing)

4.3 Study Enrolment: Patient Assent Form

Patient Assent Form

(15- to 17-year-old participants)

Protocol Title: Trauma Training Effectiveness Research Network

Principal Investigator: Name

1. **What we wish to tell you?**

I, Dr/Mr/Ms ______________________ wanted to tell you something we are doing called a research study. A research study is when doctors collect a lot of information to learn something about health and diseases.

You are being invited to be part of a research study. We will explain about the study to you and we will ask you if you would like to be part of the study. You will be given a copy of this document to take home with you.

2. **Why are we doing this study?**

We want to find out if a training program for doctors on trauma life support will help patients with injury. For this we will collect information from people who are injured like you.

3. **What will happen to you if you are in this study?**

If you agree to be part of the study, we collect data about your health available in the hospital. We will also contact you in person or by telephone to collect information about your health at the following times:

- On arrival at the emergency department and wards till you are discharged
- On hospital discharge
- 24 hours after arrival to the hospital
- 30 days after arrival to the hospital

During this time, we will also ask you details about your health and general information on returning back to your normal life and experience of the injury in your life.

4. **Is this bad or hurtful for you to be part of this study?**

No, there will be no pain or risk involved in participating in this study.

5. **How will this research be useful to you?**

This study will not make you get well. But the doctors may find out something about the how the training program can improve the care of other injured patients like you or if you are injured again.

6. **Will everybody come to know about your health condition?**

We will not tell other people that you are in this research and we will also not share information about you to anyone who does not work in the research study. We will combine the information from all the patients who agree to be part of this study and no one will be able to identify you from the combined information.

7. **Do you get anything for being in the research?**

No, you will not receive anything for being part of this study

8. **Will you tell me the results?**

The information we collect from you and other patients will be combined and studied by doctors and other researchers who are part of the study. We will publish the results in medical and scientific journals so that the knowledge from this study can help injured patients across India and the world. But no one will be able to identify you or your information in these published results.

9. **Do you have any questions?**

You can ask questions at any time. You can ask now or can ask later. You can talk to me or you can talk to someone else. I have attached the details of the person supervising the study at this hospital in case you want to contact us.

10. **Do you have to be in this study?**

No, you do not. No one will be force you if you don't want to do this. And remember, you can say yes now and change your mind later. It is up to you. You can do that at any time using the contact information provided below. This will not in any way affect your treatment in this hospital.

11. **Who can you talk to or ask questions to?**

You can also talk to anyone you want to about this like a family member, friend, or teacher. Doctor. I have attached the details of the person supervising the study at this hospital in case you want to contact us.

Name
Designation, Department
Phone Number
Email

You can also talk to anyone you want to about this like a family member, friend, or teacher. Doctor.

12. **Signature of Person Conducting Assent Discussion**

I have explained the study to ______________________________________ in language he/she can understand, and he/she has **agreed/not agreed** to be in the study.

____

(Name of the person conducting assent discussion)

___________________________________ ___________________

(Signature of the person conducting assent discussion) (Date of signing)
