# Supplementary material for: A pilot multicentre cluster randomised trial to compare the effect of trauma life support training programmes on patient and provider outcomes
Source: BMJ Open. 2022 Apr 15;12(4):e057504. doi: 10.1136/bmjopen-2021-057504 (PMC9016405; doi:10.1136/bmjopen-2021-057504)
Supplement: Supplementary data [file bmjopen-2021-057504supp002.pdf]

## Supplemental Material 2

*2.1 Study Enrolment: Resident Information Sheet*

### Participant Information Sheet

You are being invited to participate in a research study being conducted at your hospital. Please read carefully the information provided here. If you agree to participate, please sign the informed consent form. You will be given a copy of this document to keep with you.

## Protocol Title: Trauma Training Effectiveness Research Network

**Principal Investigator**: Name

**PURPOSE OF THE RESEARCH STUDY**

We are currently conducting research in this hospital to study the feasibility of assessing the effect of trauma life support training programs on care and outcome of patients with injury. Life support training, which involves skills in how to take care of injured patients when they come to hospital, may improve how well patients recover from their injuries and we are studying if, and to what extent, that is true. To better measure the outcomes of the training program at your hospital on surgical residents undergoing, we would want to measure your knowledge and confidence during the course of the study. We ask you to participate in this study because you trained at this hospital as part of the study.

**STUDY PROCEDURES**

If you agree to participate you will be provided training in a specific trauma training program as per the randomization process. This could be Advanced Trauma Life Support (ATLS) and the Primary Trauma Care (PTC) course or standard care. We will collect information related to your demography, academic background and training, as well as measure your perception of improvement in knowledge, skills, and confidence at specific points before, during, and after the training. The data collected will be confidential and anonymous.

The results of the study may be used for research that can be published as scientific articles; however, it will not be possible to identify you by reading any article that may result from this data bank. Further, data from this project will be combined with data from other hospitals that use the same system and shared for other researchers and individuals to use, but it will not be possible to identify you using that data. Research on the data without identifiers may seek to answer other questions than those stated above.

**WITHDRAWAL FROM STUDY**

Participation in this study is completely voluntary. Even if you agree to participate now you are free to withdraw at any time without giving any reason for doing so. To withdraw you contact any of the study contact persons on the numbers or emails listed below.

**POSSIBLE RISKS, DISCOMFORTS AND INCONVENIENCES**

We have not been able to identify any major risks associated with participating in this study. If you would at that point, or any other point of time, wish to withdraw from participating in the study, you are free to do so.

**POTENTIAL BENEFITS**

This research may help to improve the implementation of trauma life support training as well as improve the care of injured patients. Although you will not directly benefit from this study, your participation will contribute to medical knowledge about the effect of training surgical residents in trauma life support training programs to improve trauma care management in India.

**CONFIDENTIALITY OF STUDY AND MEDICAL RECORDS**

The results of this research may be published as a scientific article; however, it will not be possible to identify you by reading any article that may result from this work. Further, data from this project will be combined with data from other hospitals that use the same system and shared for other researchers and individuals to use, but it will not be possible to identify you using that data.

Also, Regulatory Agencies, Institutional Review Board and Ministry of Health will be granted direct access to the data collected as part of this study to check study procedures, without making any of the data public. By signing the Informed Consent Form attached, you are authorizing such access.

**COSTS OF PARTICIPATION**

No charge will be levied on you if you take part in this study. You will not receive any compensation for participating in this study.

**RESEARCH RELATED INJURY AND COMPENSATION**

Due to the observational nature of this study, it is unlikely to cause any research related injury.

**WHOM TO CONTACT IF YOU HAVE QUESTIONS**

If you have questions about this research study and your rights during the course of this study, you may contact:

Name
Department
Phone Number
Email

*2.2 Study Enrolment: Resident Consent Form*

## Consent Form

## <u>Protocol Title:</u> Trauma Training Effectiveness Research Network

**Subject Details**

Name: NRIC/PNR/SSN No.:

Address:

Date of birth ________________ Phone No:

dd/mm/yyyy

I, ________________________________________ **agree / do not agree** to participate in the project as described and, on the terms detailed in the Participant Information Sheet. The nature of my participation in the proposed project has been explained to me in ________________ by Dr/Mr/Ms ______________________. I have fully discussed and understood the purpose and procedures of this project. I have been given the Participant Information Sheet and the opportunity to ask questions about this project and have received satisfactory answers and information. I understand that my participation is voluntary and that I am free to withdraw at any time, without giving any reasons. I also give permission to the data collected form me to be used for this project. In any event of publication and sharing of the data with other researchers and individuals, I understand that this information will not bear my name or other identifiers and that due care will be taken to preserve the confidentiality of this information.

____________________________________ __________________________

(Signature/Thumbprint (Right / Left) of Subject) (Date of signing)
