# Supplementary material for: A pilot multicentre cluster randomised trial to compare the effect of trauma life support training programmes on patient and provider outcomes
Source: BMJ Open. 2022 Apr 15;12(4):e057504. doi: 10.1136/bmjopen-2021-057504 (PMC9016405; doi:10.1136/bmjopen-2021-057504)
Supplement: Supplementary data [file bmjopen-2021-057504supp003.pdf]

Supplemental Material 3

Table S3.1: Participant outcomes

Type	Outcome	Specific measurement variable	Analysis metric	Method of aggregation	Time point
Primary	All cause mortality within 30 days from the time of arrival to the emergency department	Death as reported in patient records or by a patient contact person during telephone follow up.	Final value / Change from baseline	Proportion / Difference in proportions between baseline and after intervention.	30 days from the time of arrival to the emergency department.
Secondary	All cause mortality within 24 hours from the time of arrival to the emergency department	Death as reported in patient records or by a patient contact person during telephone follow up.	Final value / Change from baseline	Proportion / Difference in proportions between baseline and after intervention.	24 hours from the time of arrival to the emergency department.
Secondary	Time to all cause mortality during follow up	Time to (in days) death as reported in patient records or by a patient contact person during telephone follow up.	Final value / Change from baseline	Survival analysis, hazard.	End of follow up.
Secondary	Cause-specific in-hospital mortality	Categorical presumed cause of death as judged by the treating physician. Recorded by asking the physician.	Final value / Change from baseline	Proportion / Difference in proportions between baseline and after intervention.	End of follow up.
Secondary	Adherence to the WHO trauma care checklist*	The number of items in the WHO trauma care checklist* that is adhered to during initial management. Recorded through observation.	Final value / Change from baseline	Mean / median.	On first encounter with surgical unit in the emergency department.

Gerdin Wärnberg M, *et al. BMJ Open* 2022; 12:e057504. doi: 10.1136/bmjopen-2021-057504

Table S3.1: Participant outcomes *(continued)*

Type	Outcome	Specific measurement variable	Analysis metric	Method of aggregation	Time point
Secondary	Fluids for resuscitation in first one hour in patients	The type of fluids, i.e. crystalloids, colloids or blood products, used in the first hour after arrival to the emergency department. Recorded through observation.	Final value / Change from baseline	Proportion.	During the first hour after the patient arrived at the emergency department.
Secondary	Massive transfusion, defined as four or more units of packed red blood cells, plasma or platelets transfused within the first 24 hours after arrival to the emergency department	The number of units of packed red blood cells, plasma or platelets transfused during the first 24 hours after the patient arrived at the emergency department. Extracted from patient records.	Final value / Change from baseline.	Proportion.	24 hours from the time of arrival to the emergency department.
Secondary	Time to first surgery	The time, in hours, from the patient's first encounter with the surgical unit, to start of surgery. Extracted from patient records.	Time to event.	Survival analysis, hazard.	24 hours from the time of arrival to the emergency department.
Secondary	Time to first intubation	The time, in hours, from the patient's first encounter with the surgical unit, to intubation. Recorded through observation.	Time to event.	Survival analysis, hazard.	24 hours from the time of arrival to the emergency department.

Table S3.1: Participant outcomes *(continued)*

Type	Outcome	Specific measurement variable	Analysis metric	Method of aggregation	Time point
Secondary	Time to CT scan	The time, in hours, from the patient's first encounter with the surgical unit, to CT scan. Extracted from patient records.	Time to event.	Survival analysis, hazard.	24 hours from the time of arrival to the emergency department.
Secondary	Ventilator free days	The number of days, out of the total length of hospital stay, that the patient is not mechanically ventilated. Extracted from patient records.	Final value / Change from baseline	Mean / median.	At patient discharge.
Secondary	ICU free days	The number of days, out of the total length of hospital stay, that the patient is not admitted to the ICU. Extracted from patient records.	Final value / Change from baseline	Mean / median.	On patient discharge.
Secondary	Pulmonary complications	Measured by identifying new infil- trates/consolidations on X-ray chest or CT-scan chest or diagnosed by a clinician or re-intubated after initially extubated.	Final value / Change from baseline.	Proportion.	On patient discharge or 30 days from the time of arrival to the emergency department, whichever occurs first.

Table S3.1: Participant outcomes *(continued)*

Type	Outcome	Specific measurement variable	Analysis metric	Method of aggregation	Time point
Secondary	Septic shock	Measured by recognizing patients needing vasopressor support beyond the first 48 hours or new initiation of vasopressors in the absence of bleeding or diagnosed by a clinician.	Final value / Change from baseline.	Proportion.	On patient discharge or 30 days from the time of arrival to the emergency department, whichever occurs first.
Secondary	Renal failure	Measured by identifying a patient on dialysis or diagnosed by a clinician.	Final value / Change from baseline.	Proportion.	On patient discharge or 30 days from the time of arrival to the emergency department, whichever occurs first.
Secondary	Coagulopathy	Measured by transfusion of plasma /platelets	Final value / Change from baseline.	Proportion.	On patient discharge or 30 days from the time of arrival to the emergency department, whichever occurs first.
Secondary	Length of stay	The number of days that the patient is admitted to the hospital. Extracted from patient records.	Final value / Change from baseline	Mean / median.	On patient discharge.

Gerdin Wärnberg M, *et al. BMJ Open* 2022; 12:e057504. doi: 10.1136/bmjopen-2021-057504

Table S3.1: Participant outcomes *(continued)*

Type	Outcome	Specific measurement variable	Analysis metric	Method of aggregation	Time point
Secondary	Quality of life	Measured using the appropriate translation of EQ5D3L. Recorded through interview or telephone follow up.	Final value / Change from baseline	Proportion or mean/median depending on domain.	30 days from the time of arrival to the emergency department.
Secondary	Number of hospitalizations after the index admission during the follow up period	The number of hospitalizations after the first (index) admission. Recorded from patient or patient contact person during telephone follow up.	Final value / Change from baseline	Mean / median.	30 days from the time of arrival to the emergency department.
Secondary	Return to work	Return to any form of work (including house work), as yes or no. Recorded through interview or telephone follow up.	Final value / Change from baseline	Proportion.	30 days from the time of arrival to the emergency department.
Secondary	Need for unplanned re-exploration	New unplanned surgery for a previously operated injury during the index admission. Extracted from patient records.	Final value / Change from baseline	Proportion.	30 days from the time of arrival to the emergency department.

Gerdin Wärnberg M, *et al. BMJ Open* 2022; 12:e057504. doi: 10.1136/bmjopen-2021-057504

Table S3.1: Participant outcomes *(continued)*

Type	Outcome	Specific measurement variable	Analysis metric	Method of aggregation	Time point
Secondary	Failure of non-operative management	Surgery for initially non-operatively treated conditions, for example liver or splenic injury in stable patients. Extracted from patient records.	Final value / Change from baseline	Proportion.	48 hours.
Secondary	Patient satisfaction	Patient satisfaction measured in Likert scale of 1-5 from Not satisfied to Satisfied completely about their hospital experience that includes healthcare person's behaviour and care received (from Harris et al. 2007)].	Final value / Change from baseline	Median	Prior to discharge
Secondary	Out-of-pocket expenditure	Direct out-of-pocket expenditure (in Indian Rupees, INR) on medicines, diagnostics, medical equipment, and follow-up treatment recorded through interview or telephone follow-up.	Final value / Change from baseline	Mean/Median	At patient discharge and 30 days from the time of arrival to the emergency department

Gerdin Wärnberg M, *et al. BMJ Open* 2022; 12:e057504. doi: 10.1136/bmjopen-2021-057504

Table S3.1: Participant outcomes *(continued)*

Type	Outcome	Specific measurement variable	Analysis metric	Method of aggregation	Time point
Secondary	Self-ambulatory	Whether or not the patient can walk unassisted. Recorded through interview or telephone follow up.	Final value / Change from baseline	Proportion.	30 days from the time of arrival to the emergency department.
Secondary	Residents' confidence in managing trauma patients	Visual Analogue Scale. Recorded through interview.	Final value / Change from baseline.	Median.	30 days from the time of training, or study start.

Note:
* The World Health Organization's trauma care checklist is available from https://www.who.int/publications/i/item/trauma-care-checklist and its implementation was published as Lashoher A, Schneider EB, Juillard C, Stevens K, Colantuoni E, Berry WR, Bloem C, Chadbunchachai W, Dharap S, Dy SM, Dziekan G, Gruen RL, Henry JA, Huwer C, Joshipura M, Kelley E, Krug E, Kumar V, Kyamanywa P, Mefire AC, Musafir M, Nathens AB, Ngendahayo E, Nguyen TS, Roy N, Pronovost PJ, Khan IQ, Razzak JA, Rubiano AM, Turner JA, Varghese M, Zakirova R, Mock C. Implementation of the World Health Organization Trauma Care Checklist Program in 11 Centers Across Multiple Economic Strata: Effect on Care Process Measures. World J Surg. 2017 Apr;41(4):954-962. doi: 10.1007/s00268-016-3759-8. PMID: 27800590.
