# Supplementary material for: A pilot multicentre cluster randomised trial to compare the effect of trauma life support training programmes on patient and provider outcomes
Source: BMJ Open. 2022 Apr 15;12(4):e057504. doi: 10.1136/bmjopen-2021-057504 (PMC9016405; doi:10.1136/bmjopen-2021-057504)
Supplement: Supplementary data [file bmjopen-2021-057504supp004.pdf]

Supplemental Material 4

Table S4.1: Variable list.

Sr No	Variable	Description
1	patient__id	Increment ID in local data base, created by reg__hospital__id, user__id and incremental value
2	local__patient__id	Hospitals patient ID to track the patient in local charts, not uploaded.
3	reg__hospital__id	Assigned ID for the hospital where regitration is taking place
4	referral	If patient was refered from another hospital
5	ref__hospital__code	Type of hospital refered from Public or Private or charitable
6	pt__age	Age of the patient
7	pt__gender	Gender of patient
8	moi	As defined in ICD-10 codes
9	dominating__injury__type	Indication of the type of injury produced by the trauma.
10	arrival__by	How did the patient arrive to hospital? (Walking, private car, EMS?)
11	ed__hr	Heart rate recorded in the emergency department.
12	ed__sbp	Systolic blood pressure in the emergency department.
13	ed__dbp	Diastolic blood pressure in the emergency department.
14	ed__gcs	Total GCS score recorded in the emergency department.
15	ed__rr	Respiratory rate recorded in the emergency department.
16	ed__sat	Saturation recorded in the emergency department.
17	ed__temperature	Temperature of patient in the emergency department.
18	ed__pupils	Pupillary response (Unilateral response, bilateral response, non-responsive unilateral or bilateral)
19	ed__shock__index	hr divided by sbp in the emergency department
20	ed__initial__serum__lactate	Serum lactate as measured in the emergency department(from ABG)
21	ed__intial__be	BE as measured in the emergency department (from ABG)
22	intubation	Endotracheal intubation done Before Arrival/After arrival/No
23	time__of__intubation	Date + time of intubation (Set to 0000-00-00 if unknown, null if not intubated)
24	time__mechanical__ventilation__started	Timestamp, coded 0000-00-00 if unknown time, null if patient was not on mechanical ventilation
25	time__mechanical__ventilated__stopped	Timestamp, coded 0000-00-00 if unknown time, null if patient was not on mechanical ventilation
26	chest__tube	Time of insertion of Intercostal drain Before Arrival/After arrival/No
27	time__of__chest__tube	Date + time of intubation (Set to 0000-00-00 if unknown, null if not placed)
28	vasopressors	Yes/No

Gerdin Wärnberg M, *et al. BMJ Open* 2022; 12:e057504. doi: 10.1136/bmjopen-2021-057504

Table S4.1: Variable list. *(continued)*

Sr No	Variable	Description
29	time_of_vasopressors	Date + time of intubation (Set to 0000-00-00 if unknown, null if not placed)
30	num_blood transfusion_within_24h	Number of transfusions given within first 24h
31	fluids_within_24h	Quantity of fluids in the first 24 hrs (other than blood)
32	intervention	Other intervention, not defined, free-text (surgical airway, packing of wound, central line, closed reduction)
33	data_of_injury	Date and time when the accident occured.
34	date_of_transport	Date and time when the EMS service started transportation from the scene, if applicable.
35	date_of_arrival	Date and time of arrival to the emergency department.
36	admitted	If the patient was admitted to hospital
37	date_of_admission	Date and time of admission to the emergency department.
38	surgery_during_stay	Yes/No
39	date_of_surgery	Date and time when the patient was taken to surgery
40	date_of_admission_icu	The time the patient was admitted to the ICU
41	date_of_admission_ward	The time the patient was admitted to the ward
42	date_of_discharge_icu	The time the patient was discharged from the ICU
43	date_of_discharge_ward	The time the patient was discharged from the ward
44	dialysis_within_30_days	Did the patient undergo dialysis during the visit
45	discharge_alive	Yes/No
46	alive_after_30_days	If a 30 day follow up is done, this can be added.
47	time_of_death	Time of death if the patient died in ED or during hospital stay or after discharge
48	type_of_initial_surgery	Free text about the surgery
49	sbp_at_start_of_surgery	First sbp recorded at start of surgery
50	time_surgery_start	Time and date when surgery started
51	time_surgery_end	Time and date when surgery ended
52	findings_or	Findings of during surgery, free text
53	injury_first_or_icd10	ICD10 codes for found injuries on first surgery
54	initial_xray_findings	First X-ray findings
55	time_fast	Time and date when FAST was done
56	findings_fast	Findings on FAST
57	time_first_ct	Time and date when the first CT was done
58	type_first_ct	Type of CT, head, abdomen
59	findings_first_ct	Findings on first CT scan
60	injury_first_ct_icd10	ICD10 code of the injuries found on first CT
61	time_second_ct	Time and date when the second CT was done
62	type_second_ct	Type of CT, head, abdomen
63	findings_second_ct	Findings on second CT scan
64	injury_second_ct_icd10	ICD10 code of the injuries found on second CT
65	findings_additional_ct	Collected findings on following CTs

Gerdin Wärnberg M, *et al. BMJ Open* 2022; 12:e057504. doi: 10.1136/bmjopen-2021-057504

Table S4.1: Variable list. *(continued)*

Sr No	Variable	Description
66	injury_following_ct	ICD10 code of the injuries found on following CTs
67	injury_external_1	Description of found external injuries
68	injury_external_1_icd10	ICD10 codes for external injuries
69	body_surface_burn	Burns over body in percent
70	inhalation_injury	If there are any inhalational burnsYes/no
71	co_morbidity_index	CCI Charlson Comorbidity Index
72	occupation	Indicate patient's usual or principal work or business to earn a living
73	prior_facility_interventions	Interventions (procedures, medications, diagnostics) administered in a facility prior to arrival at current facility
74	number_of_serious_injuries	Total number of serious injuries as judged by provider
75	physician_likely_cause _death	Likely cause of death as per the treating doctor
76	cause_of_death	Patient's official (legal) cause of death
77	complication_pulmonary	Measured by identifying new infiltrates/consolidations on X-ray chest or CT scan report suggestive of ARDS, Pneumonia or PTE or diagnosed by a clinician. Patients on mechanical ventilation: Increased FIo2, PEEP
78	pulmonary complication_reason	
79	complication_septic_shock	Patients needing inotropic support (dopamine >5 microgram/min/ NA/ Vasopressin) beyond the first 48 hours or new initiation of inotropes in absence of bleeding or diagnosed by a clinician.
80	septic shock_reason	
81	complication_renal_failure	Measured by identifying a patient on dialysis or other renal replacement therapy
82	renal_failure_reason	
83	complication_Coagulopathy	Measured by transfusion of plasma /platelets Or deranged INR and low platelets
84	quality_of_life	EQ5D & EQ5D Y (for <18 years) questionnaire at discharge and at one month post discharge
85	number_of_hospitalizations_for_this_injury	Count of no of re-hospitalisations (other hospitals or same hospitals) after discharge at home (not transfer)
86	return_to_work	Return to any kind of work not necessarily same as pre injury
87	need_for_reexploration_or_resurgery	Need for resurgery for the same region for complication or missed injury
88	failure_of_conservative_management	Failure of conservative treatment which later needed intervention (radiological/vascular or surgical)
89	patient_satisfaction	This will be an ordinal scale recording of the overall satisfaction the patient had after discharge.
90	cost_of_treatment	Direct out-of-pocket costs for treatment to the patient including medicines, diagnostics, equipment, etc.

Table S4.1: Variable list. *(continued)*

Sr No	Variable	Description
91	selfambulatory__at__discharge	Was the patient able to ambulate on his own at discharge,
92	residents__confidence__in__managing__trauma__patients	
