# Supplementary material for: A pilot multicentre cluster randomised trial to compare the effect of trauma life support training programmes on patient and provider outcomes
Source: BMJ Open. 2022 Apr 15;12(4):e057504. doi: 10.1136/bmjopen-2021-057504 (PMC9016405; doi:10.1136/bmjopen-2021-057504)
Supplement: Supplementary data [file bmjopen-2021-057504supp006.pdf]

Supplemental Material 6

6.1 Interview: Non-patient participant Information Sheet

Participant Information Sheet

You are being invited to participate in a research study. Before you take part in this research study, the study must be explained to you and you must be given the chance to ask questions. Please read carefully the information provided here. If you agree to participate, please sign the informed consent form. You will be given a copy of this document to keep with you.

Protocol Title: Trauma Training Effectiveness Research Network

Principal Investigator: Name

PURPOSE OF THE RESEARCH STUDY
We are currently conducting research in this hospital to study the feasibility of assessing the effect of trauma life support training programs on care and outcome of patients with injury. Trauma means when a person has injuries that are serious. To select the outcomes most relevant to patients we want to know what are the challenges patients face in returning back to normal life after the injury.

STUDY PROCEDURES
If you agree to participate, we will visit you at a time convenient to you to talk to you about challenges trauma patients face. You do not have to answer certain questions if you prefer not to. You can also end the interview whenever you want, even if all questions have not been answered. An audio recording of the interview may be taken.

WITHDRAWAL FROM STUDY
Participation in this study is completely voluntary. Even if you agree to participate now you are free to withdraw at any time without giving any reason for doing so. To withdraw you contact any of the study contact persons on the numbers or emails listed below.

POSSIBLE RISKS, DISCOMFORTS AND INCONVENIENCES
We have not been able to identify any major risks associated with participating in this study. If you would at that point, or any other point of time, wish to withdraw from the study, you are free to do so.

POTENTIAL BENEFITS
Our research may help to study the most relevant outcomes for patients to get back to normal life after the injury. There is no assurance you will benefit from this study. However, your participation may contribute to the medical knowledge about the effect of the use of trauma life support training programs on the life of injured patients.

PARTICIPANT'S RIGHTS

Your participation in this study is entirely voluntary. Your questions will be answered clearly and to your satisfaction. In the event of any new information becoming available that may be relevant to your willingness to continue in this study, you will be informed in a timely manner by the Principal Investigator or his/her representative.

CONFIDENTIALITY OF THE STUDY
The results of this research may be published as a scientific article; however, it will not be possible to identify you by reading any article that may result from this work. Further, data from this project will be shared with other researchers in India and abroad, but it will not be possible to identify you using only that data.

COSTS OF PARTICIPATION
If you take part in this study, there will be no charge levied on you. You will not receive any compensation for participating in this study.

RESEARCH RELATED INJURY AND COMPENSATION
The study being observational is not likely to cause any research related injury.

WHOM TO CONTACT IF YOU HAVE QUESTIONS
If you have questions about this research study and your rights during the course of this study, you may contact:

Name

Designation, Department

Phone Number

Email

6.2 Interview: Non-patient participant Consent Form

Consent Form

Protocol Title: Trauma Training Effectiveness Research Network

Subject's Particulars

Name:

Address:

Date of birth ________________ Phone No

(dd/mm/yyyy)

I, ___________________________________ **agree / do not agree** to participate in the research study as described and on the terms set out in the Participant Information Sheet. The nature of my participation in the proposed research study has been explained to me by Dr/Mr/Ms _______________________I have fully discussed and understood the purpose and procedures of this study. I have been given the Participant Information Sheet and the opportunity to ask questions about this study and have received satisfactory answers and information.

I understand that my participation is voluntary and that I am free to withdraw at any time, without giving any reasons.

In any event of publication, I understand that it will not bear my name or other identifiers and that due care will be taken to preserve the confidentiality of this information.

____________________________________ __________________________

[Signature/Thumbprint (Right / Left) of participant] (Date of signing)
