# Supplementary material for: A pilot multicentre cluster randomised trial to compare the effect of trauma life support training programmes on patient and provider outcomes
Source: BMJ Open. 2022 Apr 15;12(4):e057504. doi: 10.1136/bmjopen-2021-057504 (PMC9016405; doi:10.1136/bmjopen-2021-057504)
Supplement: Supplementary data [file bmjopen-2021-057504supp007.pdf]

Supplemental Material 7

Table S7.1: Shows which outcomes that will be assessed in which subgroups.

	All patients	Men	Women	Blunt multisystem	Penetrating	Shock	Severe traumatic brain injury	Elderly
All cause mortality within 30 days from the time of arrival to the emergency department	Yes	Yes	Yes	Yes	Yes	Yes	Yes	Yes
All cause mortality within 24 hours from the time of arrival to the emergency department	Yes	Yes	Yes	Yes	Yes	Yes	Yes	Yes
Time to all cause mortality during follow up.	Yes	Yes	Yes	Yes	Yes	Yes	Yes	Yes
Cause-specific in-hospital mortality.	Yes	Yes	Yes	Yes	Yes	Yes	Yes	Yes
Adherence to the WHO trauma care checklist.	Yes	Yes	Yes	Yes	Yes	Yes	Yes	Yes
Fluids for resuscitation in first one hour in patients.	Yes	No	No	No	No	Yes	No	No

Gerdin Wärnberg M, *et al. BMJ Open* 2022; 12:e057504. doi: 10.1136/bmjopen-2021-057504

Table S7.1: Shows which outcomes that will be assessed in which subgroups. *(continued)*

	All patients	Men	Women	Blunt multisystem	Penetrating	Shock	Severe traumatic brain injury	Elderly
Massive transfusion, defined as four or more units of packed red blood cells, plasma or platelets transfused within the first 24 hours after arrival to the emergency department.	Yes	No	No	No	No	Yes	No	No
Time to first surgery.	Yes	No	No	No	No	Yes	No	No
Time to first intubation.	Yes	No	No	No	No	No	No	No
Time to CT scan.	Yes	No	No	No	No	No	No	No
Ventilator free days.	Yes	No	No	No	No	No	No	No
ICU free days.	Yes	No	No	No	No	No	No	No
Pulmonary complications.	Yes	No	No	No	No	No	No	No
Septic shock.	Yes	No	No	No	No	No	No	No
Renal failure.	Yes	No	No	No	No	No	No	No
Coagulopathy.	Yes	No	No	No	No	No	No	No
Length of stay.	Yes	No	No	No	No	No	No	No
Quality of life.	Yes	No	No	No	No	No	No	No
Number of hospitalizations after the index admission during the follow up period.	Yes	No	No	No	No	No	No	No
Return to work.	Yes	Yes	Yes	Yes	Yes	Yes	Yes	No

Table S7.1: Shows which outcomes that will be assessed in which subgroups. *(continued)*

	All patients	Men	Women	Blunt multisystem	Penetrating	Shock	Severe traumatic brain injury	Elderly
Need for unplanned re-exploration.	Yes	No	No	No	No	No	No	No
Failure of non-operative management.	Yes	No	No	No	No	No	No	No
Patient satisfaction.	Yes	Yes	Yes	Yes	No	No	No	No
Out-of-pocked expenditure.	Yes	No	No	No	No	No	No	No
Self-ambulatory.	Yes	No	No	No	No	No	No	No

Gerdin Wärnberg M, *et al. BMJ Open* 2022; 12:e057504. doi: 10.1136/bmjopen-2021-057504
