# Supplementary material for: A pilot multicentre cluster randomised trial to compare the effect of trauma life support training programmes on patient and provider outcomes
Source: BMJ Open. 2022 Apr 15;12(4):e057504. doi: 10.1136/bmjopen-2021-057504 (PMC9016405; doi:10.1136/bmjopen-2021-057504)
Supplement: Author's manuscript [file bmjopen-2021-057504.draft_revisions.pdf]

BMJ Open is committed to open peer review. As part of this commitment we make the peer review history of every article we publish publicly available.

When an article is published we post the peer reviewers' comments and the authors' responses online. We also post the versions of the paper that were used during peer review. These are the versions that the peer review comments apply to.

The versions of the paper that follow are the versions that were submitted during the peer review process. They are not the versions of record or the final published versions. They should not be cited or distributed as the published version of this manuscript.

BMJ Open is an open access journal and the full, final, typeset and author-corrected version of record of the manuscript is available on our site with no access controls, subscription charges or pay-per-view fees (http://bmjopen.bmj.com).

If you have any questions on BMJ Open's open peer review process please email info.bmjopen@bmj.com

BMJ Open

A Pilot Multi-center Cluster Randomized Trial to Compare The Effect of Trauma Life Support Training Programs on Patient and Provider Outcomes

Journal:	*BMJ Open*
Manuscript ID	bmjopen-2021-057504
Article Type:	Protocol
Date Submitted by the Author:	17-Sep-2021

Complete List of Authors:	Gerdin Wärnberg, Martin ; Karolinska Institutet, Department of Public Health Sciences<br>Berg, Johanna; Karolinska Institutet, Department of Global Public Health; Skåne University Hospital, Emergency Medicine, Department of Internal and Emergency Medicine<br>Bhandarkar, Prashant; Bhabha Atomic Research Centre, WHO Collaborating Centre (WHOCC) for Research in Surgical Needs in LMICs; Tata Institute of Social Sciences School of Health Systems Studies,<br>Chintamani, Chintamani; Vardhman Mahavir Medical College and Safdarjung Hospital, Department of Surgery<br>Felländer-Tsai, Li; Karolinska Universitetssjukhuset Ortopedkliniken,<br>Gadgil, Anita; World Health Organization Collaborating Center for Research in Surgical Care Delivery in Low-and-Middle Income Countries<br>Ghag, Geeta; HBT Medical College And Dr. R N Cooper Municipal General Hospital, Department of Surgery<br>Hasselberg , Marie; Karolinska Institute, Global Public Health<br>Juillard, Catherine; David Geffen School of Medicine at UCLA, Division of General Surgery, Department of Surgery<br>Khajanchi, Monty; Seth Gowardhandas Sunderdas Medical College and King Edward Memorial Hospital, Department of Surgery<br>Kizhakke Veetil, Deepa; Manipal Hospital Dwarka, Department of Surgery<br>Kumar, Vineet; Lokmanya Tilak Municipal Medical College and General Hospital, Surgery<br>Mishra, Anurag; Maulana Azad Medical College,<br>Patil, Priti; Bhabha Atomic Research Centre Medical Division, Department of Statistics<br>Roy, Nobhojit; Karolinska Institutet, Dept of Global Public Health; HBNI, Dept of Surgery<br>Siddarth, David; Karolinska Institutet, Department of Public Health Sciences<br>Singh, Rajdeep; Maulana Azad Medical College, Surgery<br>Solomon, Harris; Duke University, Department of Cultural Anthropology and the Duke Global Health Institute<br>Soni, Kapil; All India Institute of Medical Sciences, Critical Care<br>Strömmer, Lovisa; Capio S:t Görans Hospital, Department of Surgery; Karolinska Institutet, Division of Surgery, Department of Clinical Science, Intervention and Technology (CLINTEC)<br>Tandon, Megha ; Vardhman Mahavir Medical College and Safdarjung

	Hospital, Department of Surgery
Keywords:	Trauma management < ORTHOPAEDIC & TRAUMA SURGERY, ACCIDENT & EMERGENCY MEDICINE, EDUCATION & TRAINING (see Medical Education & Training)

SCHOLARONE™
Manuscripts

A Pilot Multi-center Cluster Randomized Trial to Compare The Effect of Trauma Life Support Training Programs on Patient and Provider Outcomes

Study Protocol

Trial registration

We intend to register this protocol with ClinicalTrials.gov.

Protocol version

2021-09-17

Funding

Swedish Research Council (registration number 2020-03779)

Contributors

Trauma life support Effectiveness Research Network (TERN) collaborators:

Anita Gadgil (AG)

WHO collaboration Centre for Research in Surgical Care Delivery in LMIC, India.

Anurag Mishra (AM)

Department of Surgery, Maulana Azad Medical College, New Delhi, India.

Catherine Juillard (CJ)

Division of General Surgery, Department of Surgery, David Geffen School of Medicine at UCLA, Los Angeles, California, United States of America.

Chintamani (C)

Department of Surgery, Vardhman Mahavir Medical College and Safdarjung Hospital, New Delhi, India.

Deepa Kizhakke Veetil (DKV)

Department of Surgery, Manipal Hospital, Dwarka, New Delhi.

For peer review only

Geeta Ghag (GG)

Department of Surgery, HBT Medical College And Dr. R N Cooper Municipal General Hospital, Mumbai, India.

Harris Solomon (HS)

Department of Cultural Anthropology and the Duke Global Health Institute, Duke University, Durham, USA.

Johanna Berg (JB)

Department of Global Public Health, Karolinska Institutet, Stockholm Sweden.

Emergency Medicine, Department of Internal and Emergency Medicine, Skåne University Hospital, Malmö.

Kapil Dev Soni (KDS)

Critical and Intensive Care, JPN Apex Trauma Center, All India Institute of Medical Sciences, New Delhi.

Li Felländer-Tsai (LFT)

Division of Orthopaedics and Biotechnology, Department of Clinical Science Intervention and Technology (CLINTEC), Karolinska Institutet, Stockholm, Sweden.

Department of Reconstructive Orthopedics, Karolinska University Hospital, Stockholm, Sweden.

Lovisa Strömmer (LS)

Division of Surgery, Department of Clinical Science, Intervention and Technology (CLINTEC), Karolinska Institutet, Stockholm.

Department of Surgery, Capio St Göran Hospital, Stockholm, Sweden.

Marie Hasselberg (MH)

Department of Global Public Health, Karolinska Institutet, Stockholm, Sweden.

Martin Gerdin Wärnberg (MGW)

Department of Global Public Health, Karolinska Institutet, Stockholm, Sweden.

Function Perioperative Medicine and Intensive Care, Karolinska University Hospital, Solna, Sweden.

Megha Tandon (MT)

Department of Surgery, Vardhman Mahavir Medical College and Safdarjung Hospital, New Delhi, India.

For peer review only

Monty Khajanchi (MK)

WHO Collaboration Centre for Research in Surgical Care Delivery in LMIC, India.

Seth G. S. Medical College and K.E.M. Hospital, Mumbai.

Nobhojit Roy (NR)

WHO Collaborating Center for Research on Surgical Care Delivery in LMICs, BARC Hospital, HBNI University, Mumbai, India.

Department of Global Public Health, Karolinska Institutet, Stockholm, Sweden.

Prashant Bhandarkar (PB)

WHO collaboration Centre for Research in Surgical Care Delivery in LMIC, India.

Priti Patil (PP)

WHO collaboration Centre for Research in Surgical Care Delivery in LMIC, India.

Rajdeep Singh (RS)

Department of Surgery, Maulana Azad Medical College, New Delhi, India.

Siddarth David (SD)

Department of Global Public Health, Karolinska Institutet, Stockholm, Sweden.

Doctors For You, Mumbai, India.

Vineet Kumar (VK)

Department of Surgery, Lokmanya Tilak Municipal Medical College and General Hospital, Mumbai, Maharashtra, India.

Contributions

MGW conceived of the study. AG, AM, CJ, DKV, HS, JB, KDS, LFT, LS, MH, MGW, MK, NR, PB, PP, RS, SD, and VK contributed to the design of the study. DKV, KDS, MK, and MGW drafted the first version of the protocol. AG, HS, and SD drafted the first version of the patient and public involvement activities. JB and PP drafted the first versions of the data management sections and wrote the data management plan. PB and PP drafted the first versions of the statistical analysis section. AG, AM, CJ, DKV, HS, JB, KDS, LFT, LS, MH, MGW, MK, NR, PB, PP, RS, SD, and VK contributed to the refinement of the protocol. C, GG, MK, MT, and VK are representatives of prospective participating hospitals.

Role of study sponsor and funders

The funding source had no role in the design of this study and will not have any role during its execution, analyses, interpretation of the data, or decision to submit results.

For peer review only

For peer review only

Abstract

Introduction

Trauma accounts for nearly 10% of the global burden of disease. Several trauma life support programs aim to improve trauma outcomes. There is no evidence from controlled trials to show the effect of these programs on patient mortality and morbidity. Here we describe the protocol of a pilot that aims to assess the feasibility of conducting a cluster randomised controlled trial comparing Advanced Trauma Life Support (ATLS) and Primary Trauma Care (PTC) with standard care.

Methods and analysis

We will pilot a pragmatic three-armed parallel, cluster randomised, controlled trial. We will recruit Indian tertiary hospitals providing trauma care and include trauma patients and residents managing these patients. Two hospitals will be randomised to ATLS, two to PTC, and two to standard care. The primary outcome will be all cause mortality at 30 days from the time of arrival to the emergency department. Our secondary outcomes will include patient, provider, and process measures. All outcomes except time to event secondary outcomes will be measured both as final values as well as change from baseline. We will compare outcomes in three combinations of trial arms: ATLS versus PTC, ATLS versus standard care, and PTC versus standard care using absolute and relative differences along with associated confidence intervals. We will conduct subgroup analyses across clinical subgroups. In parallel to the pilot trial we will conduct community consultations to inform the planning of the larger trial.

Ethics and dissemination

We will apply for ethics approvals to the local Institutional Review Board (IRB) for each hospital as well as from the Swedish Ethical Review Authority. The protocol will be published to ClinicalTrials.gov. The results will be submitted for publication in a peer-review open-access journal. The anonymized data and code for analysis will be released publicly.

Registration details

We intend to register this protocol with ClinicalTrials.gov.

For peer review only

Article Summary

Strengths and limitations of this study:

- First controlled trial comparing ATLS , PTC and standard of care assessing effect on patient outcomes such as mortality.
- The pilot study data will help in informing future large trials.
- The cluster randomized control trial will randomize individual centers rather than individual patients thus providing more power for assessing system intervention.
- The sample size is not estimated because previous studies are lacking.
- Participating centers' heterogeneity may affect the study estimates and bias the results.

For peer review only

Introduction

Trauma, defined as the clinical entity composed of physical injury and the body's associated response, causes 4.5 millions deaths every year.[1] Almost 10% of the global burden of disease is due to trauma and trauma is the top contributor to the burden of disease in children and adults aged 10 to 49 years.[2]

Trauma care is time sensitive and early management of life or limb threatening conditions is crucial. Several trauma life support training programs have been developed to improve the early management of patients as they arrive at hospital by providing a structured framework to assessment and treatment.[3–5]

The proprietary Advanced Trauma Life Support (ATLS) is the most established trauma life support training program and more than one million doctors in over 80 countries have been trained in the program.[6] Uptake in low- and middle income countries (LMIC) has been slow, potentially due to high costs.[5]

The free Primary Trauma Care (PTC) program is the most widely spread alternative program. The goal of PTC is to improve trauma care in LMIC.[7] Like ATLS, doctors in over 80 countries have been trained in PTC, and the program has been endorsed by the World Health Organization (WHO), among other international organizations including several professional societies.[7]

Despite the widespread use of these training programs there are no controlled trials showing that they impact patient outcomes.[3–5] But there is level 1 evidence that these programs improve provider skills and practices,[8,9] and observational data suggesting that they also improve patient outcomes.[10]

We will perform a pilot study that aims to assess the feasibility of conducting a cluster randomised controlled trial comparing ATLS and PTC with standard care. Recent methodological guidelines indicate that the design of efficient cluster randomised controlled trials requires data on probable or target effect sizes, proportion of participants with the outcome (if binary), and the intracluster correlation coefficient.[11] The objectives of this pilot study will be to:

- Estimate probable effect sizes on patient outcomes associated with ATLS and PTC compared with standard care, estimate the proportion of participants with the outcome (if binary), and estimate the intracluster correlation coefficient, as a basis for future sample size calculations.
- Assess the feasibility of recruiting participants and collecting data on primary and secondary outcomes, such as mortality, in-hospital complications, length of stay, and quality of life.
- Assess how the effect sizes and directions of these effects of ATLS and PTC may differ across clinically important subgroups.

For peer review only

Methods

Trial design

This study will pilot a pragmatic three-armed parallel, cluster randomised, controlled trial. There will be two intervention arms, ATLS and PTC training, and one control arm, standard care. We will collect data for four months in all three arms, first during a one month observation phase and then during a three month intervention phase (or continued observation in the control arm). This design will allow us to assess outcomes both as final values and as change from baseline. Our study is a pilot study because its objectives involves estimating quantities, such as the probable effect sizes, proportion of participants with the outcome (if binary), and the intracluster correlation coefficient, needed for the sample size calculations of a full-scale study.[11] It will also establish how many participants that can be enrolled, as well as likely drop out rates, and the feasibility of collecting primary and secondary outcomes.

Study Setting

We will conduct this pilot in Indian tertiary hospitals. India is the world's second most populous country and has 20% of the world's trauma deaths. The trauma system is still developing, with limited prehospital care, and the in hospital trauma mortality as well as the proportion of preventable deaths remain high. Lack of standard trauma training for healthcare providers, limited hospital resources, inadequate processes of care, overcrowding emergency departments - are some of the factors that contribute to the high mortality and morbidity. During recent years efforts have been made to improve hospital trauma care, through capacity building for trained trauma care providers, augmenting facilities, and developing care protocols within the hospitals. We will conduct this trial in India because training providers in a trauma life support program is not yet the standard. Our pilot study is planned to start late 2021 or during 2022.

Eligibility Criteria for Participants and Clusters

There will be two groups of participants: patients and resident doctors.

Patient Participants

Adults (15 years or older) who present to the emergency department at participating hospitals with a history of trauma. History of trauma is here defined as having any of the external causes of morbidity and mortality listed in block V01-Y36, chapter XX of the International Classification of Disease version 10 (ICD-10) codebook as reason for presenting. We will explore intervention effects across the following clinical subgroups: men, women, blunt multisystem trauma, penetrating trauma, shock, severe traumatic brain injury, and elderly, as defined by Hornor et al.[12]

Resident Doctor Participants

Resident doctors doing their speciality training in surgery (in India it is very rare to have emergency medicine managing trauma patients in the emergency department, why we

For peer review only

decide to focus on surgical units in this pilot), who manage trauma patients in the emergency department, and who are expected to remain in the participating hospitals for at least one year. To facilitate administration each surgical department is divided into units, which manages the out patient department, emergency department, operating rooms etc on different days each week. One or two units' doctors will be selected from each hospital. One unit consists of at least three faculty and three to twelve residents.

To be eligible, units should have a maximum of 25% of the doctors trained in either ATLS, PTC, or similar training programs before the start of the pilot. Those residents who have received training in the last five years will be considered as trained. The figure of 25% was decided through consensus in the research team, to balance feasibility and contamination of results. We will select the units by conducting a prior survey to ascertain this criteria. Consent will be sought from the residents in each of the intervention groups before they undergo the ATLS or PTC training.

Clusters

Indian tertiary care hospitals that admit 400-800 adult patients with trauma each year. We randomise on the cluster (hospital) level to avoid contamination between intervention and control arms. To be eligible for inclusion hospitals have to provide the following services round the clock: operation theatres, X-ray, CT, and ultrasound facilities, and blood bank. In addition the baseline admission rate should be more than 35 adult patients with major trauma per month.

Interventions

In each intervention arm one or two units' residents per hospital providing emergency care to trauma patients will be trained in either ATLS or PTC. For the purpose of this pilot study, we will target to train a minimum of 75% of residents in each unit. If residents drop out or change units after training but before data collection is completed we will conduct additional training if needed to meet the 75% criterion. We will not train the units' faculty, as they are typically not involved in the initial management of trauma patients.

The ATLS training will be conducted in the nearest ATLS certified training centre in India according to the standard ATLS curriculum.[6] The PTC training will be arranged in hospitals randomized to the PTC arm, according to the standard PTC curriculum.[7] These courses will be conducted over a period of 2.5 to 3 days. The residents certified "pass" will be considered as trained in respective courses.

The control group provides standard care with no intervention.

Modifications

Both ATLS and PTC are standard training programs with fixed curricula.[6,7] We will not modify the delivery or content of these programs during this pilot.

For peer review only - http://bmjopen.bmj.com/site/about/guidelines.xhtml

Adherence

The intervention is the training in either ATLS or PTC. Participants are required to adhere to, i.e. participate in, the training, to be eligible for passing. We will not consider adherence to training contents during care delivery as adherence to the trial intervention, but rather as a provider level outcome.

Concomitant Care

Baseline Training

The care provided by all participating hospitals at baseline is based on the training curriculum formulated by The National Medical Council of India for post graduation in General Surgery.[13] Regarding trauma, these guidelines state that the student should:

a. Have knowledge about response to trauma; burns: causes, prevention and management; wounds of scalp and its management; recognition, diagnosis and monitoring of patients with head injury, Glasgow coma scale.
b. Be able to provide and coordinate emergency resuscitative measures in acute surgical situations including trauma.
c. Choose, perform and interpret appropriate imaging in trauma - ultrasound Focused Abdominal Sonography in Trauma (FAST).
d. Undergo advanced trauma and cardiac life support course (certified) before appearing in final examination.
e. Undergo clinical posting in emergency and trauma.
f. Present or discuss cases of blunt abdominal trauma.

Although training in an advanced trauma life support course is part of the curriculum it is optional and not doing this training does not result in failure to obtain post graduation completion.

Standard of Care

At most medical colleges in India a trauma patient comes to the emergency department where a doctor assesses the patient and refers him or her to the surgical bay for further management. In the surgical bay a second or third year general surgery resident sees all the major trauma and initiates treatment and investigations. This resident informs the consultant on call who is generally an Assistant Professor. Most procedures like intercostal drainage, open wound suturing, intubation etc. would be done in the surgical resuscitation area, by the surgical resident.

After completing the assessment and starting initial resuscitation, the resident decides to send the patient for imaging (X-rays/FAST/CT-scan) or to the operation room in consultation with or after assessment by the on-call consultant. A portable X-ray and an ultrasonography machine to conduct FAST may or may not be available in the surgical bay. The patients who are operated, managed conservatively, not intubated, or with minor trauma will be sent to the surgical ward. Those who need increased monitoring or mechanical ventilation remain in the surgical bay or in the intensive care unit (ICU)

For peer review only

depending on the availability of ICU beds. The further treatment continues in the respective ward or ICU and patients are finally discharged from the ward.

Outcomes

Our pilot study include both participant and feasibility outcomes. Prior to deciding on these participant outcomes we searched the Core Outcome Measures in Effectiveness Trials (COMET) Initiative's database but were unable to identify appropriate core outcome sets for our populations of participants.

The primary participant outcome will be all cause mortality within 30 days from the time of arrival to the emergency department. All outcomes that pertain to the individual participant level are detailed in Table 1. We decided to include a large number of outcomes, including some more exploratory, so that we can test their feasibility and relevance. We may remove secondary participant outcomes during the course of the pilot study, if they prove to be too difficult to collect. If we remove outcomes we will document the reasons for doing so.

We will also assess the following feasibility outcomes, which pertain both to overall study population as well as to the individual cluster level:

- Recruitment rate. For both patients and residents this will equal the proportion of participants enrolled, out of the total number of eligible participants, over the course of the pilot study.
- Lost to follow up rate. This will apply only to patients and equal the proportion of patients that do not complete 30 day follow up, out of all enrolled patients, over the course of the pilot study.
- Pass rate. This will apply only to residents in the intervention arms and equal the proportion of residents that pass the training programme, out of the total number of trained residents, over the course of the pilot study.
- Missing data rate. This will apply to each outcome and variable and equal the proportion of missing data, over the course of the pilot study.
- Differences in distributions of observed and extracted data. This will apply to each outcome and variable and will compare the distributions of data collected by observations versus extracted from hospital records. For quantitative variables this will be the difference in means, standard deviations, medians, interquartile ranges, and ranges. For qualitative variables this will be the differences in absolute counts and percentages, across categories.

Participant Timeline

Patients

Patients will be screened for eligibility as they arrive at the emergency department. Eligible patients will be approached in the emergency department to consent to follow up, if they are conscious. If they are unconscious a patient representative will be approached to consent to follow up. Once the patient is conscious we will approach the patient to affirm

For peer review only

the patient representative's consent. We will follow up patients at discharge, at 24 hours after arrival at the emergency department, and at 30 days after arrival at the emergency department.

Residents

Surgical units will be screened for eligibility once hospitals confirm their participation. All residents in eligible units will be approached to consent to training if their hospital is randomised to either of the intervention arms. Training will be conducted as soon as possible after the study starts. Resident participants will be followed up 30 days after training, if they are in the intervention arms, or 30 days after the study started, if they are in the control arm.

Sample size

Given budget and time constraints, including the rotation of surgical units in Indian hospitals (which often happen on a six months basis) the feasible data collection period is four months. Each of the surgical units see 2-4 trauma patients per week. If we select a minimum of one unit per hospital then each hospital will enrol 8-16 patients per month and 32-64 patients during the four months of this pilot. With a 20% attrition rate we expect each hospital to enrol 26-51 patients, coming to a total sample size of between 156 and 306 patients for this pilot study.

Recruitment

To ensure adequate recruitment we only approach hospitals with trauma volumes high enough to allow us to reach the sample size goals detailed above. Patients will be enrolled by a dedicated project officer as they arrive at the emergency department. The recruitment period will be three months. Recruitment will be monitored weekly through online conferences. No financial or non-financial incentives will be provided to trial investigators or participants for enrolment.

Allocation

Sequence generation

We will use simple randomisation to allocate sites to trial arms. We will prepare six sealed envelopes of which one representative from each pilot site will draw one. The content of the envelope will dictate what trial arm (ATLS, PTC, or standard care) the hospital will be in. There will be two hospitals in each trial arm.

Concealment Mechanism

We will not conceale the sequence, see sequence generation above.

Implementation

The random allocation sequence will be generated by the project management, who also enrol clusters. Patient participants will be included if they present during the project

For peer review only

officers shift. Resident participants are enrolled if they are in the units selected for training. We will use simple random sampling to select units if there are more than two eligible units in a hospital. For patient participants consent for follow up is sought after randomisation from patients or patient relatives as appropriate. For resident participants consent is sought before randomisation.

Blinding

It will not be possible to blind investigators or participants to interventions. We will not blind data analysts during this pilot.

Data Collection

Data collection will start one month before the training is delivered, to establish a baseline. A variability of three months of the date when data collection is started between hospitals will be accepted. Each participating hospital will have a dedicated project officer to collect data. The project officers will have a masters in a health science field and should have experience in data collection.

Because participating residents are assigned designated days for trauma care for a period of 6 months, data will be collected during those particular days and shifts when these trained doctors are in the emergency department. The project officers will collect data both by observing the care delivered and by interviewing the participants, and by extracting data from hospital records.

Data collection will continue for a minimum of three months after training. The research officers will collect data of all patients, who present with trauma in the surgical bay during their duty hours. Those patients who are admitted will be followed up for complications and other in-hospital outcome measures, for example length of stay. Patients who are not admitted will be followed up telephonically for mortality outcomes and quality of life outcomes. The follow up period will be 30 days. The project officers will make at most three attempts to reach a participant or participant representative telephonically, after which the data will be recorded as missing.

The project officer will administer the study information and informed consent (consent will only be sought for data collection including follow up) to the patient, or the patient's representative as appropriate, once the patient is stabilised. They will continue to collect data once they have received the consent.

Details of data of those patients/relatives not willing to give consent will be removed from the analysis. The number of patients who opt out from data collection will be collected, as well as limited data on their age and sex. Patients will be followed up in the ward regularly for the various outcome variables. They will also be followed up telephonically after they have been discharged.

For peer review only

Variables

The project officers will collect data on demographics, time of injury to arrival at the participating hospital, time to recording vital signs, vital signs, and times to and management details including imaging and surgery. Details of any injury sustained will be collected and coded using ICD 10 and the Abbreviated Injury Scale (AIS). For ICD 10 coders will undergo the WHO online ICD 10 training module and for AIS they will be accredited. Based on AIS we will calculate the Injury Severity Score (ISS) and the New ISS (NISS). Supplement 2 contains a full variable list, with definitions.

Patient and public involvement

In this study, inputs from patients, their caregivers, patient groups, and resident doctors will be used in the selection of outcome measures and implementation of the final study; following the Guidance for Reporting Involvement of Patients and the Public (GRIIPS 2).[14]

During the pilot study, interviews will be conducted with post-discharge trauma patients and their caregivers to identify outcomes most relevant to them. These patients will be identified through the medical registers of the participating hospitals, contacted through telephone, and after receiving their consent be interviewed as per their convenience. Additionally, members from non-government organizations working with trauma patients and the hospital Social Service Section will also be contacted for their views on contextual patient-centred outcomes for trauma patients. For feasibility, these interviews will be held in each of the cities where the participating centres are located. The commonest patient-centred outcomes reported across all the locations will be incorporated into the evaluation of the effects of the different training programs and standardized care on patient outcomes.

Similarly, the inputs of resident doctor participants at each participating centre will be collected during the pilot study. A discussion and periodic surveys will be conducted to document any challenges or suggestions they may have in the scheduling or implementation of the training programs. These inputs will be incorporated in the final study.

A summary of the findings of the study as well as their inputs will be shared with those who participated in the interviews and surveys. A meeting will be held with the patient participants, at each city, where the changes in the measured patient-centered outcomes would be presented to them. Another meeting will be held with the resident doctors at each hospital to present the confidence of the residents after being trained. Any suggestions and reflections from the participants during the meetings will be used as inputs for planning the final study.

Data management

We will supply an online data collection tool, accessible only over a virtual private network (VPN), for each participating hospital to upload pseudonymised data to secure servers. Data validation techniques like restricted values or values of a specific range will be used to avoid ambiguous data entries and ensure the validity of the data. Ambiguous responses, data errors, if any, will be resolved after discussion with the core team during weekly

For peer review only

meetings. An instruction manual or codebook for data variables will be prepared to ensure consistency in data entry. This manual will be referred to during the project data collection and variable descriptions are visible for each variable in the online data collection tool. Pseudonymised data will be stored at the centralised server. The data will be accessible by the project's principal investigator or by delegation of the project principal investigator only.

Data monitoring

Weekly meetings with the core team and project officers will take place and for this meeting a data status report will be automatically generated highlighting missing data and number of patients awaiting followup. Cluster specific interim analysis will take place after two months. The results of this will be presented to the core team, this team will decide if the pilot should be terminated. A data monitoring committee will not be used in the pilot study due to its limited scope.

Statistical Methods

We will analyse all pilot data using descriptive statistics. Quantitative variables will be summarised as mean +/- standard deviation, median, interquartile range and range. Qualitative variables will be presented as absolute numbers and percentages. Feasibility outcomes will be summarised both on the overall sample level as well as on the individual cluster level. We will use an empty generalised linear mixed model to estimate the intracluster correlation coefficient.

We will compare participant outcomes in three combinations of trial arms: ATLS versus PTC, ATLS versus standard care, and PTC versus standard care. In each combination we will compare both differences in final values and differences in change from baseline. For example, for the primary participant outcome of all cause mortality within 30 days from the time of arrival to the emergency department, comparing ATLS versus PTC, we will compare both the difference in mortality between the ATLS and PTC arms as well as the difference in the change from baseline in mortality between the ATLS and PTC arms.

For the intervention arms the change from baseline will be calculated as the difference between the one month period of data collection before the training was undertaken and the three month period after the training. For the control arm the data collection period will be four months and the difference from baseline will be calculated as the difference between the first one month and the following three months.

Within each combination of trial arms we will conduct subgroup analyses of men, women, blunt multisystem trauma, penetrating trauma, shock, severe traumatic brain injury, and elderly. Table S1.1 in Supplement 1 shows which outcomes will be assessed in which subgroups, decided through consensus in the research team. We will further compare the results of all subgroups with the results in the whole cohort, and compare the results in the female subgroup with the male subgroup, and the results in the blunt multisystem trauma subgroup with the penetrating trauma subgroup. We are aware that the numbers in some of these subgroups are likely to be small, but we include them to help guide the formulation of the statistical analysis plan for the larger trial.

For peer review only - http://bmjopen.bmj.com/site/about/guidelines.xhtml

We will calculate both absolute and relative differences for each comparison, along with 75, 85, and 95% confidence intervals. We will use an empirical bootstrap procedure with 1000 draws to estimate these confidence intervals. We will not perform any formal hypothesis tests during the analysis of this pilot's data.[15] We will also compare the data collected through observations and interviews with the data collected from hospital records, to assess the feasibility of collecting data from hospital records in the larger trial.

Ethics and Dissemination

We will apply for research ethics approval at local clusters in India to the local Institutional Review Board (IRB) committees. For managing data in Sweden ethical approval will be sought from the Swedish Ethical Review Authority. The protocol will be submitted for journal publication as well as to ClinicalTrials.gov. Amendments to the protocol after this will be determined by the core research group and updated on ClinicalTrials.gov. Substantial amendments, such as modifications to the eligibility criteria or outcomes will also be resubmitted to the journal. Declaration of interest will be submitted from all participating researchers both in the core team and at each site. The final anonymized dataset and code for analysis will be released publicly. The results will be submitted for publication in peer-review open access journals. Authorship will follow the International Committee of Medical Journal Editors (ICMJE) guidelines.

Competing Interests

Several authors are Advanced Trauma Life Support instructors.

For peer review only

Table 1. Primary and secondary participant outcomes.

Type	Outcome	Specific measurement variable	Analysis metric	Method of aggregation	Time point
Primary	All cause mortality within 30 days from the time of arrival to the emergency department	Death as reported in patient records or by a patient contact person during telephone follow up.	Final value/Change from baseline	Proportion/Difference in proportions between baseline and after intervention.	30 days from the time of arrival to the emergency department.
Secondary	All cause mortality within 24 hours from the time of arrival to the emergency department	Death as reported in patient records or by a patient contact person during telephone follow up.	Final value/Change from baseline	Proportion/Difference in proportions between baseline and after intervention.	24 hours from the time of arrival to the emergency department.
	Time to all cause mortality during follow up	Time to (in days) death as reported in patient records or by a patient contact person during telephone follow up.	Final value/Change from baseline	Survival analysis, hazard.	End of follow up.
	Cause-specific in-hospital mortality	Categorical presumed cause of death as judged by the treating physician. Recorded by asking the physician.	Final value/Change from baseline	Proportion/Difference in proportions between baseline and after intervention.	End of follow up.
	Adherence to the WHO trauma care checklist	The number of items in the WHO trauma care checklist that is adhered to during initial management. Recorded through observation.	Final value/Change from baseline	Mean/median.	On first encounter with surgical unit in the emergency department.
	Fluids for resuscitation in first one hour in patients	The type of fluids, i.e. crystalloids, colloids or	Final value/Change from baseline	Proportion.	During the first hour after the patient arrived at the

	blood products, used in the first hour after arrival to the emergency department. Recorded through observation.			emergency department.
Massive transfusion, defined as four or more units of packed red blood cells, plasma or platelets transfused within the first 24 hours after arrival to the emergency department	The number of units of packed red blood cells, plasma or platelets transfused during the first 24 hours after the patient arrived at the emergency department. Extracted from patient records.	Final value/Change from baseline.	Proportion.	24 hours from the time of arrival to the emergency department.
Time to first surgery	The time, in hours, from the patient's first encounter with the surgical unit, to start of surgery. Extracted from patient records.	Time to event.	Survival analysis, hazard.	24 hours from the time of arrival to the emergency department.
Time to first intubation	The time, in hours, from the patient's first encounter with the surgical unit, to intubation. Recorded through observation.	Time to event.	Survival analysis, hazard.	24 hours from the time of arrival to the emergency department.
Time to CT scan	The time, in hours, from the patient's first encounter with the surgical unit, to CT scan. Extracted from patient records.	Time to event.	Survival analysis, hazard.	24 hours from the time of arrival to the emergency department.

For peer review only

Ventilator free days	The number of days, out of the total length of hospital stay, that the patient is not mechanically ventilated. Extracted from patient records.	Final value/Change from baseline	Mean/median.	At patient discharge.
ICU free days	The number of days, out of the total length of hospital stay, that the patient is not admitted to the ICU. Extracted from patient records.	Final value/Change from baseline	Mean/median.	On patient discharge.
Pulmonary complications	Measured by identifying new infiltrates/consolidations on X-ray chest or CT-scan chest or diagnosed by a clinician or re-intubated after initially extubated.	Final value/Change from baseline.	Proportion.	On patient discharge or 30 days from the time of arrival to the emergency department, whichever occurs first.
Septic shock	Measured by recognizing patients needing inotropic support beyond the first 48 hours or new initiation of inotropes in absence of bleeding or diagnosed by a clinician.	Final value/Change from baseline.	Proportion.	On patient discharge or 30 days from the time of arrival to the emergency department, whichever occurs first.
Renal failure	Measured by identifying a patient on dialysis or diagnosed by a	Final value/Change from baseline.	Proportion.	On patient discharge or 30 days from the time of arrival to the

For peer review only

	clinician.			emergency department, whichever occurs first.
Coagulopathy	Measured by transfusion of plasma /platelets	Final value/Change from baseline.	Proportion.	On patient discharge or 30 days from the time of arrival to the emergency department, whichever occurs first.
Length of stay	The number of days that the patient is admitted to the hospital. Extracted from patient records.	Final value/Change from baseline	Mean/median.	On patient discharge.
Quality of life	Measured using the appropriate translation of EQ5D3L. Recorded through interview or telephone follow up.	Final value/Change from baseline	Proportion or mean/median depending on domain.	30 days from the time of arrival to the emergency department.
Number of hospitalizations after the index admission during the follow up period	The number of hospitalizations after the first (index) admission. Recorded from patient or patient contact person during telephone follow up.	Final value/Change from baseline	Mean/median.	30 days from the time of arrival to the emergency department.
Return to work	Return to any form of work (including house work), as yes or no. Recorded through interview or telephone follow up.	Final value/Change from baseline	Proportion.	30 days from the time of arrival to the emergency department.

For peer review only

Need for unplanned re-exploration	New unplanned surgery for a previously operated injury during the index admission. Extracted from patient records.	Final value/Change from baseline	Proportion.	30 days from the time of arrival to the emergency department.
Failure of conservative management	Surgery for initially conservatively treated condition, for example liver or splenic injury in stable patients. Extracted from patient records.	Final value/Change from baseline	Proportion.	48 hours.
Patient satisfaction	Patient satisfaction measured in Likert scale of 1-5 from Not satisfied to Satisfied completely about their hospital experience that includes healthcare person's behaviour and care received.[16]	Final value/Change from baseline	Median	Prior to discharge
Cost of treatment	Direct out-of-pocket expenditure (in Indian Rupees, INR) on medicines, diagnostics, medical equipment, and follow-up treatment recorded through interview or telephone	Final value/Change from baseline	Mean/Median	At patient discharge and 30 days from the time of arrival to the emergency department

For peer review only

	follow-up.			
Self-ambulatory	Whether or not the patient can walk unassisted. Recorded through interview or telephone follow up.	Final value/Change from baseline	Proportion.	30 days from the time of arrival to the emergency department.
Residents' confidence in managing trauma patients	Visual Analogue Scale. Recorded through interview.	Final value/Change from baseline.	Median.	30 days from the time of training, or study start.

References

1. Naghavi, M. *et al.* Global, regional, and national age-sex specific mortality for 264 causes of death, 1980–2016: A systematic analysis for the global burden of disease study 2016. *The Lancet* **390**, 1151–1210 (2017).

2. Vos, T. *et al.* Global burden of 369 diseases and injuries in 204 countries and territories, 1990–2019: A systematic analysis for the global burden of disease study 2019. *The Lancet* **396**, 1204–1222 (2020).

3. Mohammad, A. *et al.* Educational and clinical impact of advanced trauma life support (atls) courses: A systematic review. *World Journal of Surgery* **38**, 322–329 (2013).

4. Jayaraman, S. *et al.* Advanced trauma life support training for hospital staff. *Cochrane Database of Systematic Reviews* (2014).

5. Kadhum, M. *et al.* Are primary trauma care (ptc) courses beneficial in low- and middle-income countries - a systematic review. *Injury* **51**, 136–141 (2020).

6. American College of Surgeons, C. on T. *Advanced trauma life support® student course manual*. (American College of Surgeons, 2018).

7. Foundation, P. T. C. *Primary trauma care course manual for trauma management in locations wih limited resources*. (Primary Trauma Care Foundation, 2018).

8. Ali, J. *et al.* Demonstration of acquisition of trauma management skills by senior medical students completing the atls program. *The Journal of Trauma: Injury, Infection, and Critical Care* **38**, 687–691 (1995).

9. Ali, J. *et al.* Teaching effectiveness of the advanced trauma life support program as demonstrated by an objective structured clinical examination for practicing physicians. *World Journal of Surgery* **20**, 1121–1126 (1996).

For peer review only

10. Qin, L.-J. *et al.* Systemic analysis of pre-hospital trauma emergency treatment in zhengzhou. *Journal of Acute Disease* **8**, 34 (2019).

11. Hemming, K. *et al.* How to design efficient cluster randomised trials. *BMJ* j3064 (2017).

12. Hornor, M. A. *et al.* Quality benchmarking in trauma: From the ntdb to tqip. *Current Trauma Reports* **4**, 160–169 (2018).

13. India, M. C. of. *Guidelines for competency based postgraduate training programme for ms in general surgery*. (Medical Council of India, 2019).

14. Staniszewska, S. *et al.* GRIPP2 reporting checklists: Tools to improve reporting of patient and public involvement in research. *BMJ* j3453 (2017).

15. Lee, E. C. *et al.* The statistical interpretation of pilot trials: Should significance thresholds be reconsidered? *BMC Medical Research Methodology* **14**, (2014).

16. Harris, I. *et al.* Factors predicting patient satisfaction following major trauma. *Injury* **38**, 1102–1108 (2007).

For peer review only

Supplement 1

Table S1.1. Shows which outcomes that will be assessed in which subgroups.

	Men	Women	Blunt multisystem	Penetrating	Shock	Severe traumatic brain injury	Elderly
All cause mortality within 30 days from the time of arrival to the emergency department	Yes	Yes	Yes	Yes	Yes	Yes	Yes
All cause mortality within 24 hours from the time of arrival to the emergency department	Yes	Yes	Yes	Yes	Yes	Yes	Yes
Time to all cause mortality during follow up.	Yes	Yes	Yes	Yes	Yes	Yes	Yes
Cause-specific in-hospital mortality.	Yes	Yes	Yes	Yes	Yes	Yes	Yes
Adherence to the WHO trauma care checklist.	Yes	Yes	Yes	Yes	Yes	Yes	Yes
Fluids for resuscitation in first one hour in patients.	Yes	No	No	No	Yes	No	No
Massive transfusion, defined as four or more units of packed red blood cells, plasma or platelets transfused within the first 24 hours after arrival to the emergency department.	Yes	No	No	No	Yes	No	No
Time to first surgery.	Yes	No	No	No	Yes	No	No
Time to first intubation.	Yes	No	No	No	No	No	No
Time to CT scan.	Yes	No	No	No	No	No	No
Ventilator free days.	Yes	No	No	No	No	No	No
ICU free days.	Yes	No	No	No	No	No	No
Pulmonary complications.	Yes	No	No	No	No	No	No
Septic shock.	Yes	No	No	No	No	No	No
Renal failure.	Yes	No	No	No	No	No	No
Coagulopathy.	Yes	No	No	No	No	No	No
Length of stay.	Yes	No	No	No	No	No	No
Quality of life.	Yes	No	No	No	No	No	No
Number of hospitalizations after the index admission during the follow up period.	Yes	No	No	No	No	No	No
Return to work.	Yes	Yes	Yes	Yes	Yes	Yes	No
Need for unplanned re-exploration.	Yes	No	No	No	No	No	No

For peer review only

Failure of conservative management.	Yes	No	No	No	No	No	No
Patient satisfaction.	Yes	Yes	Yes	No	No	No	No
Cost of treatment.	Yes	No	No	No	No	No	No
Self-ambulatory.	Yes	No	No	No	No	No	No

For peer review only

Supplement 2

Table S2.1 Variable list

Sr No	Variable	Description
1	patient_id	Increment ID in local data base, created by reg_hospital_id, user_id and incremental value
2	local_patient_id	Hospitals patient ID to track the patient in local charts, not uploaded.
3	reg_hospital_id	Assigned ID for the hospital where regitration is taking place
4	referral	If patient was refered from another hospital
5	ref_hospital_code	Type of hospital refered from Public or Private or charitable
6	pt_age	Age of the patient
7	pt_gender	Gender of patient
8	moi	As defined in ICD-10 codes
9	dominating_injury_type	Indication of the type of injury produced by the trauma.
10	arrival_by	How did the patient arrive to hospital? (Walking, private car, EMS?)
11	ed_hr	Heart rate recorded in the emergency department.
12	ed_sbp	Systolic blood pressure in the emergency department.
13	ed_dbp	Diastolic blood pressure in the emergency department.
14	ed_gcs	Total GCS score recorded in the emergency department.
15	ed_rr	Respiratory rate recorded in the emergency department.
16	ed_sat	Saturation recorded in the emergency department.
17	ed_temperature	Temperature of patient in the emergency department.
18	ed_pupils	Pupillary response (Unilateral response, bilateral response, non-responsive unilateral or bilateral)
19	ed_shock_index	hr divided by sbp in the emergency department
20	ed_initial_serum_lactate	Serum lactate as measured in the emergency department(from ABG)
21	ed_intial_be	BE as measured in the emergency department (from ABG)

For peer review only

22	intubation	Endotracheal intubation done Before Arrival/After arrival/No
23	time_of_intubation	Date + time of intubation (Set to 0000-00-00 if unknown, null if not intubated)
24	time_mechanical_ventilation_started	Timestamp, coded 0000-00-00 if unknown time, null if patient was not on mechanical ventilation
25	time_mechanical_ventilated_stopped	Timestamp, coded 0000-00-00 if unknown time, null if patient was not on mechanical ventilation
26	chest_tube	Time of insertion of Intercostal drain Before Arrival/After arrival/No
27	time_of_chest_tube	Date + time of intubation (Set to 0000-00-00 if unknown, null if not placed)
28	vasopressors	Yes/No
29	time_of_vasopressors	Date + time of intubation (Set to 0000-00-00 if unknown, null if not placed)
30	num_blood transfusion_within_24h	Number of transfusions given within first 24h
31	fluids_within_24h	Quantity of fluids in the first 24 hrs (other than blood)
32	intervention	Other intervention, not defined, free-text (surgical airway, packing of wound, central line, closed reduction)
33	data_of_injury	Date and time when the accident occured.
34	date_of_transport	Date and time when the EMS service started transportation from the scene, if applicable.
35	date_of_arrival	Date and time of arrival to the emergency department.
36	admitted	If the patient was admitted to hospital
37	date_of_admission	Date and time of admission to the emergency department.
38	surgery_during_stay	Yes/No
39	date_of_surgery	Date and time when the patient was taken to surgery
40	date_of_admission_icu	The time the patient was admitted to the ICU
41	date_of_admission_ward	The time the patient was admitted to the ward
42	date_of_discharge_icu	The time the patient was discharged from the ICU
43	date_of_discharge_ward	The time the patient was discharged from the ward
44	dialysis_within_30_days	Did the patient undergo dialysis during the visit
45	discharge_alive	Yes/No
46	alive_after_30_days	If a 30 day follow up is done, this can be added.

For peer review only

47	time_of_death	Time of death if the patient died in ED or during hospital stay or after discharge
48	type_of_initial_surgery	Free text about the surgery
49	sbp_at_start_of_surgery	First sbp recorded at start of surgery
50	time_surgery_start	Time and date when surgery started
51	time_surgery_end	Time and date when surgery ended
52	findings_or	Findings of during surgery, free text
53	injury_first_or_icd10	ICD10 codes for found injuries on first surgery
54	initial_xray_findings	First X-ray findings
55	time_fast	Time and date when FAST was done
56	findings_fast	Findings on FAST
57	time_first_ct	Time and date when the first CT was done
58	type_first_ct	Type of CT, head, abdomen
59	findings_first_ct	Findings on first CT scan
60	injury_first_ct_icd10	ICD10 code of the injuries found on first CT
61	time_second_ct	Time and date when the second CT was done
62	type_second_ct	Type of CT, head, abdomen
63	findings_second_ct	Findings on second CT scan
64	injury_second_ct_icd10	ICD10 code of the injuries found on second CT
65	findings_additional_ct	Collected findings on following CTs
66	injury_following_ct	ICD10 code of the injuries found on following CTs
67	injury_external_1	Description of found external injuries
68	injury_external_1_icd10	ICD10 codes for external injuries
69	body_surface_burn	Burns over body in percent
70	inhalation_injury	If there are any inhalational burnsYes/no
71	co_morbidity_index	CCI Charlson Comorbidity Index
72	occupation	Indicate patient's usual or principal work or business to earn a living
73	prior_facility_interventions	Interventions (procedures, medications, diagnostics) administered in a facility prior to arrival at current facility
74	number_of_serious_injuries	Total number of serious injuries as judged by provider
75	physician_likely_cause _death	Likely cause of death as per the treating doctor
76	cause_of_death	Patient's official (legal) cause of death
77	complication_pulmonary	Measured by identifying new infiltrates/consolidations on X-ray chest or CT scan report suggestive of ARDS, Pneumonia or PTE or diagnosed by a clinician. Patients on mechanical ventilation: Increased FIo2, PEEP

For peer review only

78	pulmonary complication_reason	
79	complication_septic_shock	Patients needing inotropic support (dopamine >5 microgram/min/ NA/ Vasopressin) beyond the first 48 hours or new initiation of inotropes in absence of bleeding or diagnosed by a clinician.
80	septic shock_reason	
81	complication_renal_failure	Measured by identifying a patient on dialysis or other renal replacement therapy
82	renal_failure_reason	
83	complication_Coagulopathy	Measured by transfusion of plasma /platelets Or deranged INR and low platelets
84	quality_of_life	EQ5D & EQ5D Y (for <18 years) questionnaire at discharge and at one month post discharge
85	number_of_hospitalizations_for_this_injury	Count of no of re-hospitalisations (other hospitals or same hospitals) after discharge at home (not transfer)
86	return_to_work	Return to any kind of work not necessarily same as pre injury
87	need_for_reexploration_or_resurgery	Need for resurgery for the same region for complication or missed injury
88	failure_of_conservative_management	Failure of conservative treatment which later needed intervention (radiological/vascular or surgical)
89	patient_satisfaction	This will be an ordinal scale recording of the overall satisfaction the patient had after discharge.
90	cost_of_treatment	Direct out-of-pocket costs for treatment to the patient including medicines, diagnostics, equipment, etc.
91	selfambulatory_at_discharge	Was the patient able to ambulate on his own at discharge,
92	residents_confidence_in_managing_trauma_patients	

For peer review only

SPIRIT

STANDARD PROTOCOL ITEMS: RECOMMENDATIONS FOR INTERVENTIONAL TRIALS

SPIRIT 2013 Checklist: Recommended items to address in a clinical trial protocol and related documents*

Section/item	Item No	Description	Addressed on page number
**Administrative information**			
Title	1	Descriptive title identifying the study design, population, interventions, and, if applicable, trial acronym	1
Trial registration	2a	Trial identifier and registry name. If not yet registered, name of intended registry	3
	2b	All items from the World Health Organization Trial Registration Data Set	Included
Protocol version	3	Date and version identifier	1
Funding	4	Sources and types of financial, material, and other support	1
Roles and responsibilities	5a	Names, affiliations, and roles of protocol contributors	1 - 3
	5b	Name and contact information for the trial sponsor	
	5c	Role of study sponsor and funders, if any, in study design; collection, management, analysis, and interpretation of data; writing of the report; and the decision to submit the report for publication, including whether they will have ultimate authority over any of these activities	1
	5d	Composition, roles, and responsibilities of the coordinating centre, steering committee, endpoint adjudication committee, data management team, and other individuals or groups overseeing the trial, if applicable (see Item 21a for data monitoring committee)	Not applicable

Introduction

Background and rationale	6a	Description of research question and justification for undertaking the trial, including summary of relevant studies (published and unpublished) examining benefits and harms for each intervention	6
	6b	Explanation for choice of comparators	6
Objectives	7	Specific objectives or hypotheses	6
Trial design	8	Description of trial design including type of trial (eg, parallel group, crossover, factorial, single group), allocation ratio, and framework (eg, superiority, equivalence, noninferiority, exploratory)	7

Methods: Participants, interventions, and outcomes

Study setting	9	Description of study settings (eg, community clinic, academic hospital) and list of countries where data will be collected. Reference to where list of study sites can be obtained	7
Eligibility criteria	10	Inclusion and exclusion criteria for participants. If applicable, eligibility criteria for study centres and individuals who will perform the interventions (eg, surgeons, psychotherapists)	7
Interventions	11a	Interventions for each group with sufficient detail to allow replication, including how and when they will be administered	8
	11b	Criteria for discontinuing or modifying allocated interventions for a given trial participant (eg, drug dose change in response to harms, participant request, or improving/worsening disease)	8
	11c	Strategies to improve adherence to intervention protocols, and any procedures for monitoring adherence (eg, drug tablet return, laboratory tests)	8
	11d	Relevant concomitant care and interventions that are permitted or prohibited during the trial	9
Outcomes	12	Primary, secondary, and other outcomes, including the specific measurement variable (eg, systolic blood pressure), analysis metric (eg, change from baseline, final value, time to event), method of aggregation (eg, median, proportion), and time point for each outcome. Explanation of the clinical relevance of chosen efficacy and harm outcomes is strongly recommended	9-10, 15-20
Participant timeline	13	Time schedule of enrolment, interventions (including any run-ins and washouts), assessments, and visits for participants. A schematic diagram is highly recommended (see Figure)	10

Sample size	14	Estimated number of participants needed to achieve study objectives and how it was determined, including clinical and statistical assumptions supporting any sample size calculations	10
Recruitment	15	Strategies for achieving adequate participant enrolment to reach target sample size	10

Methods: Assignment of interventions (for controlled trials)

Allocation:			
Sequence generation	16a	Method of generating the allocation sequence (eg, computer-generated random numbers), and list of any factors for stratification. To reduce predictability of a random sequence, details of any planned restriction (eg, blocking) should be provided in a separate document that is unavailable to those who enrol participants or assign interventions	11
Allocation concealment mechanism	16b	Mechanism of implementing the allocation sequence (eg, central telephone; sequentially numbered, opaque, sealed envelopes), describing any steps to conceal the sequence until interventions are assigned	11
Implementation	16c	Who will generate the allocation sequence, who will enrol participants, and who will assign participants to interventions	11
Blinding (masking)	17a	Who will be blinded after assignment to interventions (eg, trial participants, care providers, outcome assessors, data analysts), and how	11
	17b	If blinded, circumstances under which unblinding is permissible, and procedure for revealing a participant's allocated intervention during the trial	11

Methods: Data collection, management, and analysis

Data collection methods	18a	Plans for assessment and collection of outcome, baseline, and other trial data, including any related processes to promote data quality (eg, duplicate measurements, training of assessors) and a description of study instruments (eg, questionnaires, laboratory tests) along with their reliability and validity, if known. Reference to where data collection forms can be found, if not in the protocol	11-12
	18b	Plans to promote participant retention and complete follow-up, including list of any outcome data to be collected for participants who discontinue or deviate from intervention protocols	11-12

Data management	19	Plans for data entry, coding, security, and storage, including any related processes to promote data quality (eg, double data entry; range checks for data values). Reference to where details of data management procedures can be found, if not in the protocol	11 - 12
Statistical methods	20a	Statistical methods for analysing primary and secondary outcomes. Reference to where other details of the statistical analysis plan can be found, if not in the protocol	13 - 14
	20b	Methods for any additional analyses (eg, subgroup and adjusted analyses)	13 - 14
	20c	Definition of analysis population relating to protocol non-adherence (eg, as randomised analysis), and any statistical methods to handle missing data (eg, multiple imputation)	13 - 14
**Methods: Monitoring**			
Data monitoring	21a	Composition of data monitoring committee (DMC); summary of its role and reporting structure; statement of whether it is independent from the sponsor and competing interests; and reference to where further details about its charter can be found, if not in the protocol. Alternatively, an explanation of why a DMC is not needed	13
	21b	Description of any interim analyses and stopping guidelines, including who will have access to these interim results and make the final decision to terminate the trial	13
Harms	22	Plans for collecting, assessing, reporting, and managing solicited and spontaneously reported adverse events and other unintended effects of trial interventions or trial conduct	Not applicable
Auditing	23	Frequency and procedures for auditing trial conduct, if any, and whether the process will be independent from investigators and the sponsor	Not applicable
**Ethics and dissemination**			
Research ethics approval	24	Plans for seeking research ethics committee/institutional review board (REC/IRB) approval	14
Protocol amendments	25	Plans for communicating important protocol modifications (eg, changes to eligibility criteria, outcomes, analyses) to relevant parties (eg, investigators, REC/IRBs, trial participants, trial registries, journals, regulators)	14

Consent or assent	26a	Who will obtain informed consent or assent from potential trial participants or authorised surrogates, and how (see Item 32)	12
	26b	Additional consent provisions for collection and use of participant data and biological specimens in ancillary studies, if applicable	Not applicable
Confidentiality	27	How personal information about potential and enrolled participants will be collected, shared, and maintained in order to protect confidentiality before, during, and after the trial	13
Declaration of interests	28	Financial and other competing interests for principal investigators for the overall trial and each study site	14
Access to data	29	Statement of who will have access to the final trial dataset, and disclosure of contractual agreements that limit such access for investigators	14
Ancillary and post-trial care	30	Provisions, if any, for ancillary and post-trial care, and for compensation to those who suffer harm from trial participation	Not applicable
Dissemination policy	31a	Plans for investigators and sponsor to communicate trial results to participants, healthcare professionals, the public, and other relevant groups (eg, via publication, reporting in results databases, or other data sharing arrangements), including any publication restrictions	14
	31b	Authorship eligibility guidelines and any intended use of professional writers	14
	31c	Plans, if any, for granting public access to the full protocol, participant-level dataset, and statistical code	14
**Appendices**			
Informed consent materials	32	Model consent form and other related documentation given to participants and authorised surrogates	
Biological specimens	33	Plans for collection, laboratory evaluation, and storage of biological specimens for genetic or molecular analysis in the current trial and for future use in ancillary studies, if applicable	Not applicable

*It is strongly recommended that this checklist be read in conjunction with the SPIRIT 2013 Explanation & Elaboration for important clarification on the items. Amendments to the protocol should be tracked and dated. The SPIRIT checklist is copyrighted by the SPIRIT Group under the Creative Commons "Attribution-NonCommercial-NoDerivs 3.0 Unported" license.

BMJ Open

A Pilot Multicenter Cluster Randomized Trial to Compare The Effect of Trauma Life Support Training Programs on Patient and Provider Outcomes

Journal:	*BMJ Open*
Manuscript ID	bmjopen-2021-057504.R1
Article Type:	Protocol
Date Submitted by the Author:	12-Mar-2022
Complete List of Authors:	Gerdin Wärnberg, Martin ; Karolinska Institutet, Department of Public Health Sciences<br>Berg, Johanna; Karolinska Institutet, Department of Global Public Health; Skåne University Hospital, Emergency Medicine, Department of Internal and Emergency Medicine<br>Bhandarkar, Prashant; Bhabha Atomic Research Centre, WHO Collaborating Centre (WHOCC) for Research in Surgical Needs in LMICs; Tata Institute of Social Sciences School of Health Systems Studies,<br>Chatterjee, Anirban; Medica Superspecialty Hospital, Department of Orthopaedic Sciences<br>Chatterjee, Shamita; Seth Sukhlal Karnani Memorial Hospital, Department of Surgery<br>Chintamani, Chintamani; Vardhman Mahavir Medical College and Safdarjung Hospital, Department of Surgery<br>Felländer-Tsai, Li; Karolinska Institutet, Division of Orthopaedics and Biotechnology, Department of Clinical Science Intervention and Technology (CLINTEC); Karolinska University Hospital, Department of Reconstructive Orthopedics<br>Gadgil, Anita; World Health Organization Collaborating Center for Research in Surgical Care Delivery in Low-and-Middle Income Countries<br>Ghag, Geeta; HBT Medical College And Dr. R N Cooper Municipal General Hospital, Department of Surgery<br>Hasselberg , Marie; Karolinska Institute, Global Public Health<br>Juillard, Catherine; David Geffen School of Medicine at UCLA, Division of General Surgery, Department of Surgery<br>Khajanchi, Monty; Seth Gowardhandas Sunderdas Medical College and King Edward Memorial Hospital, Department of Surgery<br>Kizhakke Veetil, Deepa; Manipal Hospital Dwarka, Department of Surgery<br>Kumar, Vineet; Lokmanya Tilak Municipal Medical College and General Hospital, Surgery<br>Kundu, Debabrata; Medical College Kolkata, Department of Surgery<br>Mishra, Anurag; Maulana Azad Medical College,<br>Patil, Priti; Bhabha Atomic Research Centre Medical Division, Department of Statistics<br>Roy, Nobhojit; Karolinska Institutet, Dept of Global Public Health; HBNI, Dept of Surgery<br>Roy, Amit; Sir Nil Ratan Sircar Medical College & Hospital, Department of Surgery

	Siddarth, David; Karolinska Institutet, Department of Public Health Sciences<br>Singh, Rajdeep; Maulana Azad Medical College, Surgery<br>Solomon, Harris; Duke University, Department of Cultural Anthropology and the Duke Global Health Institute<br>Soni, Kapil; All India Institute of Medical Sciences, Critical Care<br>Strömmer, Lovisa; Capio S:t Görans Hospital, Department of Surgery; Karolinska Institutet, Division of Surgery, Department of Clinical Science, Intervention and Technology (CLINTEC)<br>Tandon, Megha ; Vardhman Mahavir Medical College and Safdarjung Hospital, Department of Surgery
**Primary Subject Heading**:	Emergency medicine
Secondary Subject Heading:	Anaesthesia, Surgery
Keywords:	Trauma management < ORTHOPAEDIC & TRAUMA SURGERY, ACCIDENT & EMERGENCY MEDICINE, EDUCATION & TRAINING (see Medical Education & Training)

SCHOLARONE™
Manuscripts

A Pilot Multicenter Cluster Randomized Trial to Compare The Effect of Trauma Life Support Training Programs on Patient and Provider Outcomes

Study Protocol

Trauma life support training Effectiveness Research Network (TERN) collaborators

For peer review only

Trial registration

We intend to register this protocol with Clinical Trials Registry - India and ClinicalTrials.gov.

Protocol version

2022-02-18

Corresponding author

Martin Gerdin Wärnberg (MGW)

Department of Global Public Health
Karolinska Institutet
171 77 Stockholm
Sweden

martin.gerdin@ki.se
+46 708 53 95 98 (mobile)

Contributors

Trauma life support Effectiveness Research Network (TERN) collaborators:

Martin Gerdin Wärnberg (MGW)

Department of Global Public Health, Karolinska Institutet, Stockholm, Sweden.

Function Perioperative Medicine and Intensive Care, Karolinska University Hospital, Solna, Sweden.

Johanna Berg (JB)

Department of Global Public Health, Karolinska Institutet, Stockholm, Sweden.

Emergency Medicine, Department of Internal and Emergency Medicine, Skåne University Hospital, Malmö, Sweden.

Prashant Bhandarkar (PB)

WHO collaboration Centre for Research in Surgical Care Delivery in LMIC, Mumbai, India.

Anirban Chatterjee (AC)

Department of Orthopaedic Sciences, Medica Superspecialty Hospital, Kolkata, India.

Shamita Chatterjee (SC)

Department of Surgery, Institute of Post Graduate Medical Education and Research, Kolkata, India.

Chintamani (C)

Department of Surgery, Vardhman Mahavir Medical College and Safdarjung Hospital, New Delhi, India.

Li Felländer-Tsai (LFT)

Division of Orthopaedics and Biotechnology, Department of Clinical Science Intervention and Technology (CLINTEC), Karolinska Institutet, Stockholm, Sweden.

Department of Reconstructive Orthopedics, Karolinska University Hospital, Stockholm, Sweden.

Anita Gadgil (AG)

WHO collaboration Centre for Research in Surgical Care Delivery in LMIC, Mumbai, India.

Geeta Ghag (GG)

Department of Surgery, HBT Medical College And Dr. R N Cooper Municipal General Hospital, Mumbai, India.

Marie Hasselberg (MH)

Department of Global Public Health, Karolinska Institutet, Stockholm, Sweden.

Catherine Juillard (CJ)

Division of General Surgery, Department of Surgery, David Geffen School of Medicine at UCLA, Los Angeles, California, United States of America.

Monty Khajanchi (MK)

WHO Collaboration Centre for Research in Surgical Care Delivery in LMIC, Mumbai, India.

Seth G. S. Medical College and K.E.M. Hospital, Mumbai, India.

Deepa Kizhakke Veetil (DKV)

Department of Surgery, Manipal Hospital, Dwarka, New Delhi.

For peer review only

Vineet Kumar (VK)

Department of Surgery, Lokmanya Tilak Municipal Medical College and General Hospital, Mumbai, India.

Debabrata Kundu (DK)

Department of Surgery, Medical College Kolkata, Kolkata, India.

Anurag Mishra (AM)

Department of Surgery, Maulana Azad Medical College, New Delhi, India.

Priti Patil (PP)

WHO collaboration Centre for Research in Surgical Care Delivery in LMIC, Mumbai, India.

Nobhojit Roy (NR)

WHO Collaborating Center for Research on Surgical Care Delivery in LMICs, BARC Hospital, HBNI University, Mumbai, India.

Department of Global Public Health, Karolinska Institutet, Stockholm, Sweden.

Amit Roy (AR)

Department of Surgery, Sir Nil Ratan Sircar Medical College & Hospital, Kolkata, India.

Siddarth David (SD)

Department of Global Public Health, Karolinska Institutet, Stockholm, Sweden.

Doctors For You, Mumbai, India.

Rajdeep Singh (RS)

Department of Surgery, Maulana Azad Medical College, New Delhi, India.

Harris Solomon (HS)

Department of Cultural Anthropology and the Duke Global Health Institute, Duke University, Durham, USA.

Kapil Dev Soni (KDS)

Critical and Intensive Care, JPN Apex Trauma Center, All India Institute of Medical Sciences, New Delhi, India.

Lovisa Strömmer (LS)

Division of Surgery, Department of Clinical Science, Intervention and Technology (CLINTEC), Karolinska Institutet, Stockholm, Sweden.

For peer review only

Department of Surgery, Capio St Göran Hospital, Stockholm, Sweden.

Megha Tandon (MT)

Department of Surgery, Vardhman Mahavir Medical College and Safdarjung Hospital, New Delhi, India.

Keywords

Trauma management, Accident and emergency medicine, Education and training.

Word Count

4494

Role of study sponsor and funders

The funding sources had no role in the design of this study and will not have any role during its execution, analyses, interpretation of the data, or decision to submit results.

For peer review only

Abstract

Introduction Trauma accounts for nearly 10% of the global burden of disease. Several trauma life support programs aim to improve trauma outcomes. There is no evidence from controlled trials to show the effect of these programs on patient outcomes. We describe the protocol of a pilot study that aims to assess the feasibility of conducting a cluster randomised controlled trial comparing Advanced Trauma Life Support (ATLS) and Primary Trauma Care (PTC) with standard care. **Methods and analysis** We will pilot a pragmatic three-armed parallel, cluster randomised, controlled trial in India, where neither of these programs are routinely taught. We will recruit tertiary hospitals and include trauma patients and residents managing these patients. Two hospitals will be randomised to ATLS, two to PTC, and two to standard care. The primary outcome will be all cause mortality at 30 days from the time of arrival to the emergency department. Our secondary outcomes will include patient, provider, and process measures. All outcomes except time to event outcomes will be measured both as final values as well as change from baseline. We will compare outcomes in three combinations of trial arms: ATLS versus PTC, ATLS versus standard care, and PTC versus standard care using absolute and relative differences along with associated confidence intervals. We will conduct subgroup analyses across the clinical subgroups men, women, blunt multisystem trauma, penetrating trauma, shock, severe traumatic brain injury, and elderly. In parallel to the pilot study we will conduct community consultations to inform the planning of the full-scale trial. **Ethics and dissemination** We will apply for ethics approvals to the local Institutional Review Board (IRB) in each hospital. The protocol will be published to Clinical Trials Registry - India and ClinicalTrials.gov. The results will be published and the anonymized data and code for analysis will be released publicly.

For peer review only

Article Summary

Strengths and limitations of this study:

- Cluster randomized controlled trial comparing the effect of ATLS, PTC and standard care on patient and provider outcomes.
- Prospective data collection with direct observations by dedicated project officers.
- Participating centers' heterogeneity may affect the study estimates and bias the results.

For peer review only

Introduction

Trauma, defined as the clinical entity composed of physical injury and the body's associated response, causes 4.5 millions deaths every year.[1] Almost 10% of the global burden of disease is due to trauma and trauma is the top contributor to the burden of disease in children and adults aged 10 to 49 years.[2]

Trauma care is time sensitive and early management of life or limb threatening conditions is crucial. Several trauma life support training programs have been developed to improve the early management of patients as they arrive at hospital by providing a structured framework to assessment and treatment.[3–5]

The proprietary Advanced Trauma Life Support (ATLS) is the most established trauma life support training program and more than one million doctors in over 80 countries have been trained in the program.[6] Uptake in low- and middle income countries (LMIC) has been slow, potentially due to high costs.[5]

The free Primary Trauma Care (PTC) program is the most widely spread alternative program. The goal of PTC is to improve trauma care in LMIC.[7] Like ATLS, doctors in over 80 countries have been trained in PTC, and the program has been endorsed by the World Health Organization (WHO), among other international organizations including several professional societies.[7]

Despite the widespread use of these training programs there are no controlled trials showing that they impact patient outcomes.[3–5] But there is level 1 evidence that these programs improve provider skills and practices,[8,9] and observational data suggesting that they also improve patient outcomes.[10]

We will perform a pilot study that aims to assess the feasibility of conducting a cluster randomised controlled trial comparing ATLS and PTC with standard care. Recent methodological guidelines indicate that the design of efficient cluster randomised controlled trials requires data on probable or target effect sizes, proportion of participants with the outcome (if binary), and the intracluster correlation coefficient.[11] The objectives of this pilot study will be to:

- Estimate probable effect sizes on patient outcomes associated with ATLS and PTC compared with standard care, estimate the proportion of participants with the outcome (if binary), and estimate the intracluster correlation coefficient, as a basis for future sample size calculations.
- Assess the feasibility of recruiting participants and collecting data on primary and secondary outcomes, such as mortality, in-hospital complications, length of stay, and quality of life.
- Assess how the effect sizes and directions of these effects of ATLS and PTC may differ across clinically important subgroups.

For peer review only

Methods

Trial Design

This study will pilot a pragmatic three-armed parallel, cluster randomised, controlled trial. There will be two intervention arms, ATLS and PTC training, and one control arm, standard care. We will collect data for four months in all three arms, first during a one month observation phase and then during a three month intervention phase (or continued observation in the control arm). This design will allow us to assess outcomes both as final values and as change from baseline. Our study is a pilot study because its objectives involves estimating quantities, such as the probable effect sizes, proportion of participants with the outcome (if binary), and the intracluster correlation coefficient, needed for the sample size calculations of a full-scale trial.[11] The full-scale trial will be planned regardless of the effect-sizes identified in this pilot study. This pilot study will also establish how many participants that can be enrolled, as well as likely drop out rates, and the feasibility of collecting primary and secondary outcomes.

Study Setting

We will conduct this pilot in Indian tertiary hospitals, where neither ATLS, PTC, nor any other trauma life support training program is routinely taught. India is the world's second most populous country and has 20% of the world's trauma deaths. The trauma system is still developing, with limited prehospital care, and the in hospital trauma mortality as well as the proportion of preventable deaths remain high. Lack of standard trauma training for healthcare providers, limited hospital resources, inadequate processes of care, overcrowding emergency departments - are some of the factors that contribute to the high mortality and morbidity. During recent years efforts have been made to improve hospital trauma care, through capacity building for trained trauma care providers, augmenting facilities, and developing care protocols within the hospitals. We will conduct this trial in India because training providers in a trauma life support program is not yet the standard. Our pilot study is planned to start late 2021 or during 2022.

Eligibility Criteria for Participants and Clusters

There will be two groups of participants: patients and resident doctors.

Patient Participants

Adults (15 years or older) who present to the emergency department at participating hospitals with a history of trauma. History of trauma is here defined as having any of the external causes of morbidity and mortality listed in block V01-Y36, chapter XX of the International Classification of Disease version 10 (ICD-10) codebook as reason for presenting. We will explore intervention effects across the following clinical subgroups: men, women, blunt multisystem trauma, penetrating trauma, shock, severe traumatic brain injury, and elderly, as defined by Hornor et al.[12] The consent form for patients are available as Supplemental Material 1.

For peer review only

Resident Doctor Participants

Resident doctors doing their speciality training in surgery (in India it is very rare to have emergency medicine managing trauma patients in the emergency department, why we decide to focus on surgical units in this pilot), who manage trauma patients in the emergency department, and who are expected to remain in the participating hospitals for at least one year. To facilitate administration each surgical department is divided into units, which manages the out patient department, emergency department, operating rooms etc on different days each week. One or two, out of typically six, units' residents will be selected from each hospital. One unit consists of at least three faculty and three to twelve residents.

To be eligible, units should have a maximum of 25% of the doctors trained in either ATLS, PTC, or similar training programs before the start of the pilot (hospitals that have so far agreed to participate have no or single current residents trained in any program). Those residents who have received training in the last five years will be considered as trained. The figure of 25% was decided through consensus in the research team, to balance feasibility and contamination of results. We will select the units by conducting a prior survey to ascertain this criteria. Consent will be sought from the residents in each of the intervention groups before they undergo the ATLS or PTC training. The consent form for residents are available as Supplemental Material 2. We will not ask for consent from residents at the units in the control hospitals as their practice will not be affected by this pilot and we will not collect any personal identifiable data on them. This is in line with ethical regulations in the study setting.

Clusters

Indian tertiary care hospitals that admit 400-800 adult patients with trauma each year. We randomise on the cluster (hospital) level to avoid contamination between intervention and control arms. To be eligible for inclusion hospitals have to provide the following services round the clock: operation theatres, X-ray, CT, and ultrasound facilities, and blood bank. In addition the baseline admission rate should be more than 35 adult patients with major trauma per month.

Interventions

In each intervention arm one or two units', out of typically six, residents per hospital providing emergency care to trauma patients will be trained in either ATLS or PTC. For the purpose of this pilot study, we will target to train a minimum of 75% of residents in each unit. If residents drop out or change units after training but before data collection is completed we will conduct additional training if needed to meet the 75% criterion. We will not train the units' faculty, as they are typically not involved in the initial management of trauma patients.

The ATLS training will be conducted in the nearest ATLS certified training centre in India according to the standard ATLS curriculum.[6] The PTC training will be arranged in hospitals randomized to the PTC arm, according to the standard PTC curriculum.[7] These courses will

For peer review only

be conducted over a period of 2.5 to 3 days. The residents certified “pass” will be considered as trained in respective courses.

The control group provides standard care with no intervention.

Modifications

Both ATLS and PTC are standard training programs with fixed curricula.[6,7] We will not modify the delivery or content of these programs during this pilot.

Adherence

The intervention is the training in either ATLS or PTC. Participants are required to adhere to, i.e. participate in, the training, to be eligible for passing. We will not consider adherence to training contents during care delivery as adherence to the trial intervention, but rather as a provider level outcome.

Concomitant Care

Baseline Training

The care provided by all participating hospitals at baseline is based on the training curriculum formulated by The National Medical Council of India for post graduation in General Surgery.[13] Regarding trauma, these guidelines state that the student should:

a. Have knowledge about response to trauma; burns: causes, prevention and management; wounds of scalp and its management; recognition, diagnosis and monitoring of patients with head injury, Glasgow coma scale.
b. Be able to provide and coordinate emergency resuscitative measures in acute surgical situations including trauma.
c. Choose, perform and interpret appropriate imaging in trauma - ultrasound Focused Abdominal Sonography in Trauma (FAST).
d. Undergo advanced trauma and cardiac life support course (certified) before appearing in final examination.
e. Undergo clinical posting in emergency and trauma.
f. Present or discuss cases of blunt abdominal trauma.

Although training in an advanced trauma life support course is part of the curriculum it is optional and not doing this training does not result in failure to obtain post graduation completion.

Standard of Care

At most medical colleges in India trauma patients present to the emergency department where they are assessed by a doctor and referred to the surgical bay for further management. In the surgical bay a second or third year general surgery resident sees all the major trauma and provide the initial care, including initiating treatment and investigations. This resident informs the consultant on call who is generally an Assistant Professor. Most

For peer review only

procedures like intercostal drainage, open wound suturing, intubation etc. would be done in the surgical resuscitation area, by the surgical resident.

Compared to other settings where a trauma team approach is adopted, nurses and other healthcare professionals are involved to a limited extent during the initial management. Their roles include assisting during intubation and other bedside procedures, charting the vitals (not recording) and giving injections. They also accompany the resident during transfers of serious patients.

After completing the assessment and starting initial resuscitation, the resident decides to send the patient for imaging (X-rays/FAST/CT-scan) or to the operation room in consultation with or after assessment by the on-call consultant. A portable X-ray and an ultrasonography machine to conduct FAST may or may not be available in the surgical bay. The patients who are operated, managed conservatively, not intubated, or with minor trauma will be sent to the surgical ward. Those who need increased monitoring or mechanical ventilation remain in the surgical bay or in the intensive care unit (ICU) depending on the availability of ICU beds. The further treatment continues in the respective ward or ICU and patients are finally discharged from the ward.

Outcomes

Our pilot study include both participant and feasibility outcomes. Prior to deciding on these participant outcomes we searched the Core Outcome Measures in Effectiveness Trials (COMET) Initiative's database but were unable to identify appropriate core outcome sets for our populations of participants.

The primary participant outcome will be all cause mortality within 30 days from the time of arrival to the emergency department. The primary outcome and most secondary outcome will be assessed and compared both as final values and as change from baseline. All outcomes that pertain to the individual participant level are detailed in Supplemental Material 3. We decided to include a large number of outcomes, including some more exploratory, so that we can test their feasibility and relevance. We may remove secondary participant outcomes during the course of the pilot study, if they prove to be too difficult to collect. If we remove outcomes we will document the reasons for doing so.

We will also assess the following feasibility outcomes, which pertain both to overall study population as well as to the individual cluster level:

- Recruitment rate. For both patients and residents this will equal the proportion of participants enrolled, out of the total number of eligible participants, over the course of the pilot study.
- Lost to follow up rate. This will apply only to patients and equal the proportion of patients that do not complete 30 day follow up, out of all enrolled patients, over the course of the pilot study.
- Pass rate. This will apply only to residents in the intervention arms and equal the proportion of residents that pass the training programme, out of the total number of trained residents, over the course of the pilot study.

For peer review only

- Missing data rate. This will apply to each outcome and variable and equal the proportion of missing data, over the course of the pilot study.
- Differences in distributions of observed and extracted data. This will apply to each outcome and variable and will compare the distributions of data collected by observations versus extracted from hospital records. For quantitative variables this will be the difference in means, standard deviations, medians, interquartile ranges, and ranges. For qualitative variables this will be the differences in absolute counts and percentages, across categories.

Participant Timeline

Patients

Patients will be screened for eligibility as they arrive at the emergency department. Eligible patients will be approached in the emergency department to consent to follow up, if they are conscious. If they are unconscious a patient representative will be approached to consent to follow up. Once the patient is conscious we will approach the patient to affirm the patient representative's consent. We will follow up patients at discharge, at 24 hours after arrival at the emergency department, and at 30 days after arrival at the emergency department.

Residents

Surgical units will be screened for eligibility once hospitals confirm their participation. All residents in eligible units will be approached to consent to training if their hospital is randomised to either of the intervention arms. Training will be conducted as soon as possible after the study starts. Resident participants will be followed up 30 days after training, if they are in the intervention arms, or 30 days after the study started, if they are in the control arm.

Sample size

Given budget and time constraints, including the rotation of surgical units in Indian hospitals (which often happen on a six months basis) the feasible data collection period is four months. Each of the surgical units see 2-4 trauma patients per week. If we select a minimum of one unit per hospital then each hospital will enrol 8-16 patients per month and 32-64 patients during the four months of this pilot. With a 20% attrition rate we expect each hospital to enrol 26-51 patients, coming to a total sample size of between 156 and 306 patients for this pilot study.

Recruitment

To ensure adequate recruitment we only approach hospitals with trauma volumes high enough to allow us to reach the sample size goals detailed above. Patients will be enrolled by a dedicated project officer as they arrive at the emergency department. The recruitment period will be three months. Recruitment will be monitored weekly through online conferences. No financial or non-financial incentives will be provided to trial investigators or participants for enrolment.

For peer review only

Allocation

Sequence generation

We will use simple randomisation to allocate sites to trial arms. We will prepare six sealed envelopes of which one representative from each pilot site will draw one. The content of the envelope will dictate what trial arm (ATLS, PTC, or standard care) the hospital will be in. There will be two hospitals in each trial arm.

Concealment Mechanism

We will not conceal the sequence, see sequence generation above.

Implementation

The random allocation sequence will be generated by the project's core group, who also enrol clusters. Patient participants will be included if they present during the project officers shift. Resident participants are enrolled if they are in the units selected for training. We will use simple random sampling to select units if there are more than two eligible units in a hospital. For patient participants consent for follow up is sought after randomisation from patients or patient relatives as appropriate. For resident participants consent is sought before randomisation. If residents in a unit decline to participate, so that the target of training 75% of residents in a given unit cannot be met, another unit will be selected for participation.

Blinding

It will not be possible to blind investigators or participants to interventions. We will not blind the data analysts during this pilot, but we plan to blind the data analysts during the full-scale trial.

Data Collection

Data collection will start one month before the training is delivered, to establish a baseline. A variability of three months of the date when data collection is started between hospitals will be accepted. Each participating hospital will have a dedicated project officer to collect data. The project officers will have a masters in a health science field and should have experience in data collection.

Because participating residents are assigned designated days for trauma care for a period of 6 months, data will be collected during those particular days and shifts when these trained doctors are in the emergency department. The project officers will collect data both by observing the care delivered and by interviewing the participants, and by extracting data from hospital records.

Data collection will continue for a minimum of three months after training. The research officers will collect data of all patients, who present with trauma in the surgical bay during their duty hours. Those patients who are admitted will be followed up for complications and other in-hospital outcome measures, for example length of stay. Patients who are not

For peer review only

admitted will be followed up telephonically for mortality outcomes and quality of life outcomes. The follow up period will be 30 days. The project officers will make at most three attempts to reach a participant or participant representative telephonically, after which the data will be recorded as missing.

The project officer will administer the study information and informed consent (consent will only be sought for data collection including follow up) to the patient, or the patient's representative as appropriate, once the patient is stabilised. They will continue to collect data once they have received the consent.

Details of data of those patients/relatives not willing to give consent will be removed from the analysis. The number of patients who opt out from data collection will be collected, as well as limited data on their age and sex. Patients will be followed up in the ward regularly for the various outcome variables. They will also be followed up telephonically after they have been discharged.

Variables

The project officers will collect data on demographics, time of injury to arrival at the participating hospital, time to recording vital signs, vital signs, and times to and management details including imaging and surgery. Details of any injury sustained will be collected and coded using ICD 10 and the Abbreviated Injury Scale (AIS). For ICD 10 coders will undergo the WHO online ICD 10 training module and for AIS they will be accredited. Based on AIS we will calculate the Injury Severity Score (ISS) and the New ISS (NISS). Supplemental Material 4 contains a full variable list, with definitions.

Patient and public involvement

In this study, we will conduct community consultations to collect inputs from patients, their caregivers, patient groups, and resident doctors to be used in the selection of outcome measures and implementation of the full-scale trial; following the Guidance for Reporting Involvement of Patients and the Public (GRIIPS 2).[14]

During the pilot study, interviews will be conducted with post-discharge trauma patients and their caregivers to identify outcomes most relevant to them. These patients will be identified through the medical registers of the participating hospitals, contacted through telephone, and after receiving their consent be interviewed as per their convenience. Their consent form is available as Supplemental Material 5. Additionally, members from non-government organizations working with trauma patients and the hospital Social Service Section will also be contacted for their views on contextual patient-centred outcomes for trauma patients. Their consent form is available as Supplemental Material 6. For feasibility, these interviews will be held in each of the cities where the participating centres are located. The commonest patient-centred outcomes reported across all the locations will be incorporated into the evaluation of the effects of the different training programs and standardized care on patient outcomes.

Similarly, the inputs of resident doctor participants at each participating centre will be collected during the pilot study. A discussion and periodic surveys will be conducted to

For peer review only

document any challenges or suggestions they may have in the scheduling or implementation of the training programs. These inputs will be incorporated in the final study.

A summary of the findings of the study as well as their inputs will be shared with those who participated in the interviews and surveys. A meeting will be held with the patient participants, at each city, where the changes in the measured patient-centered outcomes would be presented to them. Another meeting will be held with the resident doctors at each hospital to present the confidence of the residents after being trained. Any suggestions and reflections from the participants during the meetings will be used as inputs for planning the final study.

Data management

We will supply an online data collection tool, accessible only over a virtual private network (VPN), for each participating hospital to upload pseudonymised data to secure servers. Data validation techniques like restricted values or values of a specific range will be used to avoid ambiguous data entries and ensure the validity of the data. Ambiguous responses, data errors, if any, will be resolved after discussion with the core team during weekly meetings. An instruction manual or codebook for data variables will be prepared to ensure consistency in data entry. This manual will be referred to during the project data collection and variable descriptions are visible for each variable in the online data collection tool. Pseudonymised data will be stored at the centralised server. The data will be accessible by the project's principal investigator or by delegation of the project principal investigator only.

Data monitoring

Weekly meetings with the core team and project officers will take place and for this meeting a data status report will be automatically generated highlighting missing data and number of patients awaiting followup. Cluster specific interim analysis will take place after two months. The results of this will be presented to the core team, this team will decide if the pilot should be terminated. Although we will not have formal termination criteria because of the short duration of the study, reasons not to continue could include that the collection of key variables, such as mortality outcomes, is unfeasible or that patients are not consenting to be included in the data collection. A data monitoring committee will not be used in the pilot study due to its limited scope.

Statistical Methods

We will analyse all pilot data using descriptive statistics. Quantitative variables will be summarised as mean +/- standard deviation, median, interquartile range and range. Qualitative variables will be presented as absolute numbers and percentages. Feasibility outcomes will be summarised both on the overall sample level as well as on the individual cluster level. We will use an empty generalised linear mixed model to estimate the intracluster correlation coefficient.

For peer review only

We will compare participant outcomes in three combinations of trial arms: ATLS versus PTC, ATLS versus standard care, and PTC versus standard care. In each combination we will compare both differences in final values and differences in change from baseline. For example, for the primary participant outcome of all cause mortality within 30 days from the time of arrival to the emergency department, comparing ATLS versus PTC, we will compare both the difference in mortality between the ATLS and PTC arms as well as the difference in the change from baseline in mortality between the ATLS and PTC arms.

For the intervention arms the change from baseline will be calculated as the difference between the one month period of data collection before the training was undertaken and the three month period after the training. For the control arm the data collection period will be four months and the difference from baseline will be calculated as the difference between the first one month and the following three months.

Within each combination of trial arms we will conduct subgroup analyses of men, women, blunt multisystem trauma, penetrating trauma, shock, severe traumatic brain injury, and elderly. Table S7.1 in Supplemental Material 7 shows which outcomes will be assessed in which subgroups, decided through consensus in the research team. We will further compare the results of all subgroups with the results in the whole cohort, and compare the results in the female subgroup with the male subgroup, and the results in the blunt multisystem trauma subgroup with the penetrating trauma subgroup. We are aware that the numbers in some of these subgroups are likely to be small, but we include them to help guide the formulation of the statistical analysis plan for the full-scale trial.

We will calculate both absolute and relative differences for each comparison, along with 75, 85, and 95% confidence intervals. We will use an empirical bootstrap procedure with 1000 draws to estimate these confidence intervals. We will not perform any formal hypothesis tests during the analysis of this pilot's data.[15] We will also compare the data collected through observations and interviews with the data collected from hospital records, to assess the feasibility of collecting data from hospital records in the full-scale trial.

Ethics and Dissemination

We will apply for research ethics approval at local clusters in India to the local Institutional Review Board (IRB) committees. The protocol will be submitted for journal publication as well as to Clinical Trials Registry - India and ClinicalTrials.gov. Amendments to the protocol after this will be determined by the core research group and updated on Clinical Trials Registry - India and ClinicalTrials.gov. Substantial amendments, such as modifications to the eligibility criteria or outcomes will also be resubmitted to the journal. Declaration of interest will be submitted from all participating researchers both in the core team and at each site. The final anonymized dataset and code for analysis will be released publicly. The results will be submitted for publication in peer-reviewed open access journals. Authorship will follow the International Committee of Medical Journal Editors (ICMJE) guidelines.

For peer review only

Contributorship statement

MGW conceived of the study. AG, AM, CJ, DKV, HS, JB, KDS, LFT, LS, MH, MGW, MK, NR, PB, PP, RS, SD, and VK contributed to the design of the study. DKV, KDS, MK, and MGW drafted the first version of the protocol. AG, HS, and SD drafted the first version of the patient and public involvement activities. JB and PP drafted the first versions of the data management sections and wrote the data management plan. PB and PP drafted the first versions of the statistical analysis section. AG, AM, CJ, DKV, HS, JB, KDS, LFT, LS, MH, MGW, MK, NR, PB, PP, RS, SC, SD, and VK contributed to the refinement of the protocol. AR, AC, C, DK, GG, MK, MT, and VK are representatives of prospective participating hospitals.

Competing Interests

Several authors are Advanced Trauma Life Support instructors.

Funding

Doctors for You through grants awarded to Karolinska Institutet by the Swedish Research Council (grant number 2020-03779) and the Laerdal Foundation (grant number 2021-0048).

Data Sharing Statement

The final anonymized dataset and code for analysis will be released publicly.

References

1. Naghavi, M. *et al.* Global, regional, and national age-sex specific mortality for 264 causes of death, 1980–2016: A systematic analysis for the global burden of disease study 2016. *The Lancet* **390**, 1151–1210 (2017).

2. Vos, T. *et al.* Global burden of 369 diseases and injuries in 204 countries and territories, 1990–2019: A systematic analysis for the global burden of disease study 2019. *The Lancet* **396**, 1204–1222 (2020).

3. Mohammad, A. *et al.* Educational and clinical impact of advanced trauma life support (atls) courses: A systematic review. *World Journal of Surgery* **38**, 322–329 (2013).

4. Jayaraman, S. *et al.* Advanced trauma life support training for hospital staff. *Cochrane Database of Systematic Reviews* (2014).

5. Kadhum, M. *et al.* Are primary trauma care (ptc) courses beneficial in low- and middle-income countries - a systematic review. *Injury* **51**, 136–141 (2020).

For peer review only

6. American College of Surgeons, C. on T. *Advanced trauma life support® student course manual*. (American College of Surgeons, 2018).

7. Foundation, P. T. C. *Primary trauma care course manual for trauma management in locations wih limited resources*. (Primary Trauma Care Foundation, 2018).

8. Ali, J. *et al.* Demonstration of acquisition of trauma management skills by senior medical students completing the atls program. *The Journal of Trauma: Injury, Infection, and Critical Care* **38**, 687–691 (1995).

9. Ali, J. *et al.* Teaching effectiveness of the advanced trauma life support program as demonstrated by an objective structured clinical examination for practicing physicians. *World Journal of Surgery* **20**, 1121–1126 (1996).

10. Qin, L.-J. *et al.* Systemic analysis of pre-hospital trauma emergency treatment in zhengzhou. *Journal of Acute Disease* **8**, 34 (2019).

11. Hemming, K. *et al.* How to design efficient cluster randomised trials. *BMJ* j3064 (2017).

12. Hornor, M. A. *et al.* Quality benchmarking in trauma: From the ntdb to tqip. *Current Trauma Reports* **4**, 160–169 (2018).

13. India, M. C. of. *Guidelines for competency based postgraduate training programme for ms in general surgery*. (Medical Council of India, 2019).

14. Staniszewska, S. *et al.* GRIPP2 reporting checklists: Tools to improve reporting of patient and public involvement in research. *BMJ* j3453 (2017).

15. Lee, E. C. *et al.* The statistical interpretation of pilot trials: Should significance thresholds be reconsidered? *BMC Medical Research Methodology* **14**, (2014).

For peer review only

Supplemental Material 1

1.1. Study Enrolment: Patient Information Sheet

Patient Information Sheet

You are being invited to participate in a research study. Before your data can be included in this data bank the purpose of the data collection must be explained to you, and you must be given the chance to ask questions. Please read carefully the information provided here. If you agree to participate, please sign the informed consent form. You will be given a copy of this document to take home with you.

For peer review only

<u>Protocol Title:</u> Trauma Training Effectiveness Research Network

Principal Investigator: Name

PURPOSE OF THE RESEARCH STUDY

We are currently conducting research in this hospital to study the feasibility of assessing the effect of trauma life support training programs on care and outcome of patients with injury. We ask you to participate in this study because you presented to this hospital after having an injury.

STUDY PROCEDURES AND VISIT SCHEDULE

If you agree to participate, we will:

- Store health data registered in your hospital records and vital signs recordings from the emergency department
- Contact you in person or by telephone for follow ups to obtain information about your health status at the following times:
    - On arrival at the emergency department and wards till you are discharged
    - On hospital discharge
    - 24 hours after arrival to the hospital
    - 30 days after arrival to the hospital

When you arrived at the hospital, we recorded some basic parameters such as your age, gender, and how you were injured. We also recorded health data such as blood pressure, heart rate, oxygen levels, respiratory rate, surgical care and treatment provided. During your stay, we will record periodically health data, the investigations and treatment that you have undergone. During the follow-up calls, we may ask you details about your health and general information on returning back to your normal life and experience of the injury in your life. If you want complete information regarding all the parameters that were recorded, please do not hesitate to ask, and we will be happy to inform you.

Should you wish that your data is deleted from the study, you may please tell us now or contact us using the contact information provided below. The results of the study may be used for research that can be published as scientific articles; however, it will not be possible to identify you by reading any article that may result from this data bank. Further, data from this project will be combined with data from other hospitals that use the same system and shared for other researchers and individuals to use, but it will not be possible to identify you using that data. Research on the data without identifiers may seek to answer other questions than those stated above.

WITHDRAWAL FROM STUDY

Participation in this study is completely voluntary. Even if you agree to participate now you are free to withdraw at any time without giving any reason for doing so. Withdrawing will not affect your ordinary treatment or the care given to you. To withdraw contact any of the contact persons using the contact information provided. Note that we can only delete data from the data collected in this hospital. We cannot delete data once it has been de-identified because we will not be able to tell from whom the data came.

POSSIBLE RISKS, DISCOMFORTS AND INCONVENIENCES

We have not been able to identify any major risks associated with participating in this study. Even if you agree to participate now you are free to withdraw at any time without giving any reason for doing so. You are free to withdraw at that point, or at any time using the contact information provided below.

POTENTIAL BENEFITS

This research may help to improve the care of injured patients. Although this study will not affect the care you are given in this hospital at this time, your participation will contribute to medical knowledge, and may improve care for you if you are injured again in the future care for others that are injured.

SUBJECT'S RIGHTS

Your participation in this study is entirely voluntary. Your questions will be answered clearly and to your satisfaction. In the event of any new information becoming available that may be relevant to your willingness to continue in this study, you will be informed in a timely manner by the Principal Investigator or his/her representative.

CONFIDENTIALITY OF STUDY AND MEDICAL RECORDS

The results of this research may be published as a scientific article; however, it will not be possible to identify you by reading any article that may result from this work. Further, data from this project will be combined with data from other hospitals that use the same system and shared for other researchers and individuals to use, but it will not be possible to identify you using that data.

Also, Regulatory Agencies, Institutional Review Board and Ministry of Health will be granted direct access to your original medical records to check study procedures and data, without

For peer review only

making any of your information public. By signing the Informed Consent Form attached, you are authorizing such access to your study and medical records.

COSTS OF PARTICIPATION

No charge will be levied on you if you take part in this study. You will not receive any compensation for participating in this study.

RESEARCH RELATED INJURY AND COMPENSATION

Due to the observational nature of this project, it is unlikely to cause any research related injury. The hospital will provide medical care for any problems that may arise during this study.

WHOM TO CONTACT IF YOU HAVE QUESTIONS

If you have questions regarding this study and your rights, or in the case of any injuries sustained during this project, you may contact the Principal Investigator:

Name
Designation, Department
Phone Number
Email

For peer review only

1.2 Study Enrolment: Patient Consent Form

Consent Form

Protocol Title: Trauma Training Effectiveness Research Network

Patient Details

Name: NRIC/PNR/SSN No.:

Address:

Date of birth ________________ Phone No:

dd/mm/yyyy

Phone number(s) of your relatives or friends that you agree we may contact, in case you do not answer your phone:

Part I – to be filled by the patient

I, ____ (Pt ID No.__________________) **agree / do not agree** to participate in the project as described and, on the terms detailed in the Patient Information Sheet. The nature of my participation in the proposed project has been explained to me in ________________ by Dr/Mr/Ms _______________________. I have fully discussed and understood the purpose and procedures of this project. I have been given the Patient Information Sheet and the opportunity to ask questions about this project and have received satisfactory answers and information. I understand that my participation is voluntary and that I am free to withdraw at any time, without giving any reasons and without my medical care being affected. I also give permission for information in my medical records to be used for this project. In any event of publication and sharing of the data with other researchers and individuals, I understand that this information will not bear my name or other identifiers and that due care will be taken to preserve the confidentiality of this information.

____________________________________ __________________________

(Signature/Thumbprint (Right / Left) of Subject) (Date of signing)

Part II – to be filled by parent / legal guardian, where applicable

I, ___________________________ hereby give consent for the above patient to participate in the proposed project. The nature, risks and benefits of the study have been explained clearly to me and I fully understand them.

____ __________________________

For peer review only

(Signature/Thumbprint (Right / Left) (Date of signing)

of parent /legal guardian]

Part III – to be filled witness, where applicable

An impartial witness should be present during the entire informed consent discussion if a subject or the subject's legally acceptable representative is unable to read. After the written informed consent form and any written information to be provided to subjects, is read and explained to the subject or the subject's legally acceptable representative, and after the subject or the subject's legally representative has orally consented to the subject's participation in the study and, if capable of doing so, has signed and personally dated the consent form, the witness should sign and personally date the consent form.

_______________________________ _______________________________

(Name of witness) (Designation of witness)

_______________________________ _______________________________

(Signature of witness) (Date of signing)

4.3 Study Enrolment: Patient Assent Form

For peer review only

Patient Assent Form

(15- to 17-year-old participants)

Protocol Title: Trauma Training Effectiveness Research Network

Principal Investigator: Name

1. **What we wish to tell you?**

I, Dr/Mr/Ms ______________________ wanted to tell you something we are doing called a research study. A research study is when doctors collect a lot of information to learn something about health and diseases.

You are being invited to be part of a research study. We will explain about the study to you and we will ask you if you would like to be part of the study. You will be given a copy of this document to take home with you.

2. **Why are we doing this study?**

We want to find out if a training program for doctors on trauma life support will help patients with injury. For this we will collect information from people who are injured like you.

3. **What will happen to you if you are in this study?**

If you agree to be part of the study, we collect data about your health available in the hospital. We will also contact you in person or by telephone to collect information about your health at the following times:

- On arrival at the emergency department and wards till you are discharged
- On hospital discharge
- 24 hours after arrival to the hospital
- 30 days after arrival to the hospital

During this time, we will also ask you details about your health and general information on returning back to your normal life and experience of the injury in your life.

4. **Is this bad or hurtful for you to be part of this study?**

No, there will be no pain or risk involved in participating in this study.

5. **How will this research be useful to you?**

This study will not make you get well. But the doctors may find out something about the how the training program can improve the care of other injured patients like you or if you are injured again.

For peer review only

6. Will everybody come to know about your health condition?

We will not tell other people that you are in this research and we will also not share information about you to anyone who does not work in the research study. We will combine the information from all the patients who agree to be part of this study and no one will be able to identify you from the combined information.

7. Do you get anything for being in the research?

No, you will not receive anything for being part of this study

8. Will you tell me the results?

The information we collect from you and other patients will be combined and studied by doctors and other researchers who are part of the study. We will publish the results in medical and scientific journals so that the knowledge from this study can help injured patients across India and the world. But no one will be able to identify you or your information in these published results.

9. Do you have any questions?

You can ask questions at any time. You can ask now or can ask later. You can talk to me or you can talk to someone else. I have attached the details of the person supervising the study at this hospital in case you want to contact us.

10. Do you have to be in this study?

No, you do not. No one will be force you if you don't want to do this. And remember, you can say yes now and change your mind later. It is up to you. You can do that at any time using the contact information provided below. This will not in any way affect your treatment in this hospital.

11. Who can you talk to or ask questions to?

You can also talk to anyone you want to about this like a family member, friend, or teacher. Doctor. I have attached the details of the person supervising the study at this hospital in case you want to contact us.

Name
Designation, Department
Phone Number
Email

You can also talk to anyone you want to about this like a family member, friend, or teacher. Doctor.

12. Signature of Person Conducting Assent Discussion

For peer review only

I have explained the study to ____ in language he/she can understand, and he/she has **agreed/not agreed** to be in the study.

____

(Name of the person conducting assent discussion)

____________________________________ ____________________

(Signature of the person conducting assent discussion) (Date of signing)

For peer review only

Supplemental Material 2

2.1 Study Enrolment: Resident Information Sheet

Participant Information Sheet

You are being invited to participate in a research study being conducted at your hospital. Please read carefully the information provided here. If you agree to participate, please sign the informed consent form. You will be given a copy of this document to keep with you.

Protocol Title: Trauma Training Effectiveness Research Network

Principal Investigator: Name

PURPOSE OF THE RESEARCH STUDY

We are currently conducting research in this hospital to study the feasibility of assessing the effect of trauma life support training programs on care and outcome of patients with injury. Life support training, which involves skills in how to take care of injured patients when they come to hospital, may improve how well patients recover from their injuries and we are studying if, and to what extent, that is true. To better measure the outcomes of the training program at your hospital on surgical residents undergoing, we would want to measure your knowledge and confidence during the course of the study. We ask you to participate in this study because you trained at this hospital as part of the study.

STUDY PROCEDURES

If you agree to participate you will be provided training in a specific trauma training program as per the randomization process. This could be Advanced Trauma Life Support (ATLS) and the Primary Trauma Care (PTC) course or standard care. We will collect information related to your demography, academic background and training, as well as measure your perception of improvement in knowledge, skills, and confidence at specific points before, during, and after the training. The data collected will be confidential and anonymous.

The results of the study may be used for research that can be published as scientific articles; however, it will not be possible to identify you by reading any article that may result from this data bank. Further, data from this project will be combined with data from other hospitals that use the same system and shared for other researchers and individuals to use, but it will not be possible to identify you using that data. Research on the data without identifiers may seek to answer other questions than those stated above.

WITHDRAWAL FROM STUDY

Participation in this study is completely voluntary. Even if you agree to participate now you are free to withdraw at any time without giving any reason for doing so. To withdraw you contact any of the study contact persons on the numbers or emails listed below.

POSSIBLE RISKS, DISCOMFORTS AND INCONVENIENCES

For peer review only

We have not been able to identify any major risks associated with participating in this study. If you would at that point, or any other point of time, wish to withdraw from participating in the study, you are free to do so.

POTENTIAL BENEFITS

This research may help to improve the implementation of trauma life support training as well as improve the care of injured patients. Although you will not directly benefit from this study, your participation will contribute to medical knowledge about the effect of training surgical residents in trauma life support training programs to improve trauma care management in India.

CONFIDENTIALITY OF STUDY AND MEDICAL RECORDS

The results of this research may be published as a scientific article; however, it will not be possible to identify you by reading any article that may result from this work. Further, data from this project will be combined with data from other hospitals that use the same system and shared for other researchers and individuals to use, but it will not be possible to identify you using that data.

Also, Regulatory Agencies, Institutional Review Board and Ministry of Health will be granted direct access to the data collected as part of this study to check study procedures, without making any of the data public. By signing the Informed Consent Form attached, you are authorizing such access.

COSTS OF PARTICIPATION

No charge will be levied on you if you take part in this study. You will not receive any compensation for participating in this study.

RESEARCH RELATED INJURY AND COMPENSATION

Due to the observational nature of this study, it is unlikely to cause any research related injury.

WHOM TO CONTACT IF YOU HAVE QUESTIONS

If you have questions about this research study and your rights during the course of this study, you may contact:

Name

Department

Phone Number

Email

For peer review only

2.2 Study Enrolment: Resident Consent Form

Consent Form

<u>Protocol Title:</u> Trauma Training Effectiveness Research Network

Subject Details

Name: NRIC/PNR/SSN No.:

Address:

Date of birth ________________ Phone No:

dd/mm/yyyy

I, ____ **agree / do not agree** to participate in the project as described and, on the terms detailed in the Participant Information Sheet. The nature of my participation in the proposed project has been explained to me in ________________ by Dr/Mr/Ms ______________________. I have fully discussed and understood the purpose and procedures of this project. I have been given the Participant Information Sheet and the opportunity to ask questions about this project and have received satisfactory answers and information. I understand that my participation is voluntary and that I am free to withdraw at any time, without giving any reasons. I also give permission to the data collected form me to be used for this project. In any event of publication and sharing of the data with other researchers and individuals, I understand that this information will not bear my name or other identifiers and that due care will be taken to preserve the confidentiality of this information.

____________________________________ ____________________________

(Signature/Thumbprint (Right / Left) of Subject) (Date of signing)

For peer review only

Supplemental Material 3

Table S3.1: Participant outcomes

Type	Outcome	Specific measurement variable	Analysis metric	Method of aggregation	Time point
Primary	All cause mortality within 30 days from the time of arrival to the emergency department	Death as reported in patient records or by a patient contact person during telephone follow up.	Final value / Change from baseline	Proportion / Difference in proportions between baseline and after intervention.	30 days from the time of arrival to the emergency department.
Secondary	All cause mortality within 24 hours from the time of arrival to the emergency department	Death as reported in patient records or by a patient contact person during telephone follow up.	Final value / Change from baseline	Proportion / Difference in proportions between baseline and after intervention.	24 hours from the time of arrival to the emergency department.
Secondary	Time to all cause mortality during follow up	Time to (in days) death as reported in patient records or by a patient contact person during telephone follow up.	Final value / Change from baseline	Survival analysis, hazard.	End of follow up.
Secondary	Cause-specific in-hospital mortality	Categorical presumed cause of death as judged by the treating physician. Recorded by asking the physician.	Final value / Change from baseline	Proportion / Difference in proportions between baseline and after intervention.	End of follow up.
Secondary	Adherence to the WHO trauma care checklist*	The number of items in the WHO trauma care checklist* that is adhered to during initial management. Recorded through observation.	Final value / Change from baseline	Mean / median.	On first encounter with surgical unit in the emergency department.

For peer review only - http://bmjopen.bmj.com/site/about/guidelines.xhtml

Table S3.1: Participant outcomes *(continued)*

Type	Outcome	Specific measurement variable	Analysis metric	Method of aggregation	Time point
Secondary	Fluids for resuscitation in first one hour in patients	The type of fluids, i.e. crystalloids, colloids or blood products, used in the first hour after arrival to the emergency department. Recorded through observation.	Final value / Change from baseline	Proportion.	During the first hour after the patient arrived at the emergency department.
Secondary	Massive transfusion, defined as four or more units of packed red blood cells, plasma or platelets transfused within the first 24 hours after arrival to the emergency department	The number of units of packed red blood cells, plasma or platelets transfused during the first 24 hours after the patient arrived at the emergency department. Extracted from patient records.	Final value / Change from baseline.	Proportion.	24 hours from the time of arrival to the emergency department.
Secondary	Time to first surgery	The time, in hours, from the patient's first encounter with the surgical unit, to start of surgery. Extracted from patient records.	Time to event.	Survival analysis, hazard.	24 hours from the time of arrival to the emergency department.
Secondary	Time to first intubation	The time, in hours, from the patient's first encounter with the surgical unit, to intubation. Recorded through observation.	Time to event.	Survival analysis, hazard.	24 hours from the time of arrival to the emergency department.

For peer review only

Table S3.1: Participant outcomes *(continued)*

Type	Outcome	Specific measurement variable	Analysis metric	Method of aggregation	Time point
Secondary	Time to CT scan	The time, in hours, from the patient's first encounter with the surgical unit, to CT scan. Extracted from patient records.	Time to event.	Survival analysis, hazard.	24 hours from the time of arrival to the emergency department.
Secondary	Ventilator free days	The number of days, out of the total length of hospital stay, that the patient is not mechanically ventilated. Extracted from patient records.	Final value / Change from baseline	Mean / median.	At patient discharge.
Secondary	ICU free days	The number of days, out of the total length of hospital stay, that the patient is not admitted to the ICU. Extracted from patient records.	Final value / Change from baseline	Mean / median.	On patient discharge.
Secondary	Pulmonary complications	Measured by identifying new infil-trates/consolidations on X-ray chest or CT-scan chest or diagnosed by a clinician or re-intubated after initially extubated.	Final value / Change from baseline.	Proportion.	On patient discharge or 30 days from the time of arrival to the emergency department, whichever occurs first.

For peer review only - http://bmjopen.bmj.com/site/about/guidelines.xhtml

Table S3.1: Participant outcomes *(continued)*

Type	Outcome	Specific measurement variable	Analysis metric	Method of aggregation	Time point
Secondary	Septic shock	Measured by recognizing patients needing vasopressor support beyond the first 48 hours or new initiation of vasopressors in the absence of bleeding or diagnosed by a clinician.	Final value / Change from baseline.	Proportion.	On patient discharge or 30 days from the time of arrival to the emergency department, whichever occurs first.
Secondary	Renal failure	Measured by identifying a patient on dialysis or diagnosed by a clinician.	Final value / Change from baseline.	Proportion.	On patient discharge or 30 days from the time of arrival to the emergency department, whichever occurs first.
Secondary	Coagulopathy	Measured by transfusion of plasma /platelets	Final value / Change from baseline.	Proportion.	On patient discharge or 30 days from the time of arrival to the emergency department, whichever occurs first.
Secondary	Length of stay	The number of days that the patient is admitted to the hospital. Extracted from patient records.	Final value / Change from baseline	Mean / median.	On patient discharge.

For peer review only

Table S3.1: Participant outcomes *(continued)*

Type	Outcome	Specific measurement variable	Analysis metric	Method of aggregation	Time point
Secondary	Quality of life	Measured using the appropriate translation of EQ5D3L. Recorded through interview or telephone follow up.	Final value / Change from baseline	Proportion or mean/median depending on domain.	30 days from the time of arrival to the emergency department.
Secondary	Number of hospitalizations after the index admission during the follow up period	The number of hospitalizations after the first (index) admission. Recorded from patient or patient contact person during telephone follow up.	Final value / Change from baseline	Mean / median.	30 days from the time of arrival to the emergency department.
Secondary	Return to work	Return to any form of work (including house work), as yes or no. Recorded through interview or telephone follow up.	Final value / Change from baseline	Proportion.	30 days from the time of arrival to the emergency department.
Secondary	Need for unplanned re-exploration	New unplanned surgery for a previously operated injury during the index admission. Extracted from patient records.	Final value / Change from baseline	Proportion.	30 days from the time of arrival to the emergency department.

For peer review only

Table S3.1: Participant outcomes *(continued)*

Type	Outcome	Specific measurement variable	Analysis metric	Method of aggregation	Time point
Secondary	Failure of non-operative management	Surgery for initially non-operatively treated conditions, for example liver or splenic injury in stable patients. Extracted from patient records.	Final value / Change from baseline	Proportion.	48 hours.
Secondary	Patient satisfaction	Patient satisfaction measured in Likert scale of 1-5 from Not satisfied to Satisfied completely about their hospital experience that includes healthcare person's behaviour and care received (from Harris et al. 2007)].	Final value / Change from baseline	Median	Prior to discharge
Secondary	Out-of-pocket expenditure	Direct out-of-pocket expenditure (in Indian Rupees, INR) on medicines, diagnostics, medical equipment, and follow-up treatment recorded through interview or telephone follow-up.	Final value / Change from baseline	Mean/Median	At patient discharge and 30 days from the time of arrival to the emergency department

For peer review only

Table S3.1: Participant outcomes *(continued)*

Type	Outcome	Specific measurement variable	Analysis metric	Method of aggregation	Time point
Secondary	Self-ambulatory	Whether or not the patient can walk unassisted. Recorded through interview or telephone follow up.	Final value / Change from baseline	Proportion.	30 days from the time of arrival to the emergency department.
Secondary	Residents' confidence in managing trauma patients	Visual Analogue Scale. Recorded through interview.	Final value / Change from baseline.	Median.	30 days from the time of training, or study start.

Note:
* The World Health Organization's trauma care checklist is available from https://www.who.int/publications/i/item/trauma-care-checklist and its implementation was published as Lashoher A, Schneider EB, Juillard C, Stevens K, Colantuoni E, Berry WR, Bloem C, Chadbunchachai W, Dharap S, Dy SM, Dziekan G, Gruen RL, Henry JA, Huwer C, Joshipura M, Kelley E, Krug E, Kumar V, Kyamanywa P, Mefire AC, Musafir M, Nathens AB, Ngendahayo E, Nguyen TS, Roy N, Pronovost PJ, Khan IQ, Razzak JA, Rubiano AM, Turner JA, Varghese M, Zakirova R, Mock C. Implementation of the World Health Organization Trauma Care Checklist Program in 11 Centers Across Multiple Economic Strata: Effect on Care Process Measures. World J Surg. 2017 Apr;41(4):954-962. doi: 10.1007/s00268-016-3759-8. PMID: 27800590.

For peer review only

Supplemental Material 4

Table S4.1: Variable list.

Sr No	Variable	Description
1	patient__id	Increment ID in local data base, created by reg__hospital__id, user__id and incremental value
2	local__patient__id	Hospitals patient ID to track the patient in local charts, not uploaded.
3	reg__hospital__id	Assigned ID for the hospital where regitration is taking place
4	referral	If patient was refered from another hospital
5	ref__hospital__code	Type of hospital refered from Public or Private or charitable
6	pt__age	Age of the patient
7	pt__gender	Gender of patient
8	moi	As defined in ICD-10 codes
9	dominating__injury__type	Indication of the type of injury produced by the trauma.
10	arrival__by	How did the patient arrive to hospital? (Walking, private car, EMS?)
11	ed__hr	Heart rate recorded in the emergency department.
12	ed__sbp	Systolic blood pressure in the emergency department.
13	ed__dbp	Diastolic blood pressure in the emergency department.
14	ed__gcs	Total GCS score recorded in the emergency department.
15	ed__rr	Respiratory rate recorded in the emergency department.
16	ed__sat	Saturation recorded in the emergency department.
17	ed__temperature	Temperature of patient in the emergency department.
18	ed__pupils	Pupillary response (Unilateral response, bilateral response, non-responsive unilateral or bilateral)
19	ed__shock__index	hr divided by sbp in the emergency department
20	ed__initial__serum__lactate	Serum lactate as measured in the emergency department(from ABG)
21	ed__intial__be	BE as measured in the emergency department (from ABG)
22	intubation	Endotracheal intubation done Before Arrival/After arrival/No
23	time__of__intubation	Date + time of intubation (Set to 0000-00-00 if unknown, null if not intubated)
24	time__mechanical__ventilation__started	Timestamp, coded 0000-00-00 if unknown time, null if patient was not on mechanical ventilation
25	time__mechanical__ventilated__stopped	Timestamp, coded 0000-00-00 if unknown time, null if patient was not on mechanical ventilation
26	chest__tube	Time of insertion of Intercostal drain Before Arrival/After arrival/No
27	time__of__chest__tube	Date + time of intubation (Set to 0000-00-00 if unknown, null if not placed)
28	vasopressors	Yes/No

For peer review only - http://bmjopen.bmj.com/site/about/guidelines.xhtml

Table S4.1: Variable list. *(continued)*

Sr No	Variable	Description
29	time__of__vasopressors	Date + time of intubation (Set to 0000-00-00 if unknown, null if not placed)
30	num__blood transfusion__within__24h	Number of transfusions given within first 24h
31	fluids__within__24h	Quantity of fluids in the first 24 hrs (other than blood)
32	intervention	Other intervention, not defined, free-text (surgical airway, packing of wound, central line, closed reduction)
33	data__of__injury	Date and time when the accident occured.
34	date__of__transport	Date and time when the EMS service started transportation from the scene, if applicable.
35	date__of__arrival	Date and time of arrival to the emergency department.
36	admitted	If the patient was admitted to hospital
37	date__of__admission	Date and time of admission to the emergency department.
38	surgery__during__stay	Yes/No
39	date__of__surgery	Date and time when the patient was taken to surgery
40	date__of__admission__icu	The time the patient was admitted to the ICU
41	date__of__admission__ward	The time the patient was admitted to the ward
42	date__of__discharge__icu	The time the patient was discharged from the ICU
43	date__of__discharge__ward	The time the patient was discharged from the ward
44	dialysis__within__30__days	Did the patient undergo dialysis during the visit
45	discharge__alive	Yes/No
46	alive__after__30__days	If a 30 day follow up is done, this can be added.
47	time__of__death	Time of death if the patient died in ED or during hospital stay or after discharge
48	type__of__initial__surgery	Free text about the surgery
49	sbp__at__start__of__surgery	First sbp recorded at start of surgery
50	time__surgery__start	Time and date when surgery started
51	time__surgery__end	Time and date when surgery ended
52	findings__or	Findings of during surgery, free text
53	injury__first__or__icd10	ICD10 codes for found injuries on first surgery
54	initial__xray__findings	First X-ray findings
55	time__fast	Time and date when FAST was done
56	findings__fast	Findings on FAST
57	time__first__ct	Time and date when the first CT was done
58	type__first__ct	Type of CT, head, abdomen
59	findings__first__ct	Findings on first CT scan
60	injury__first__ct__icd10	ICD10 code of the injuries found on first CT
61	time__second__ct	Time and date when the second CT was done
62	type__second__ct	Type of CT, head, abdomen
63	findings__second__ct	Findings on second CT scan
64	injury__second__ct__icd10	ICD10 code of the injuries found on second CT
65	findings__additional__ct	Collected findings on following CTs

For peer review only - http://bmjopen.bmj.com/site/about/guidelines.xhtml

Table S4.1: Variable list. *(continued)*

Sr No	Variable	Description
66	injury__following__ct	ICD10 code of the injuries found on following CTs
67	injury__external__1	Description of found external injuries
68	injury__external__1__icd10	ICD10 codes for external injuries
69	body__surface__burn	Burns over body in percent
70	inhalation__injury	If there are any inhalational burnsYes/no
71	co__morbidity__index	CCI Charlson Comorbidity Index
72	occupation	Indicate patient's usual or principal work or business to earn a living
73	prior__facility__interventions	Interventions (procedures, medications, diagnostics) administered in a facility prior to arrival at current facility
74	number__of__serious__injuries	Total number of serious injuries as judged by provider
75	physician__likely__cause __death	Likely cause of death as per the treating doctor
76	cause__of__death	Patient's official (legal) cause of death
77	complication__pulmonary	Measured by identifying new infiltrates/consolidations on X-ray chest or CT scan report suggestive of ARDS, Pneumonia or PTE or diagnosed by a clinician. Patients on mechanical ventilation: Increased FIo2, PEEP
78	pulmonary complication__reason	
79	complication__septic__shock	Patients needing inotropic support (dopamine >5 microgram/min/ NA/ Vasopressin) beyond the first 48 hours or new initiation of inotropes in absence of bleeding or diagnosed by a clinician.
80	septic shock__reason	
81	complication__renal__failure	Measured by identifying a patient on dialysis or other renal replacement therapy
82	renal__failure__reason	
83	complication__Coagulopathy	Measured by transfusion of plasma /platelets Or deranged INR and low platelets
84	quality__of__life	EQ5D & EQ5D Y (for <18 years) questionnaire at discharge and at one month post discharge
85	number__of__hospitalizations__for__this__injury	Count of no of re-hospitalisations (other hospitals or same hospitals) after discharge at home (not transfer)
86	return__to__work	Return to any kind of work not necessarily same as pre injury
87	need__for__reexploration__or__resurgery	Need for resurgery for the same region for complication or missed injury
88	failure__of__conservative__management	Failure of conservative treatment which later needed intervention (radiological/vascular or surgical)
89	patient__satisfaction	This will be an ordinal scale recording of the overall satisfaction the patient had after discharge.
90	cost__of__treatment	Direct out-of-pocket costs for treatment to the patient including medicines, diagnostics, equipment, etc.

For peer review only - http://bmjopen.bmj.com/site/about/guidelines.xhtml

Table S4.1: Variable list. *(continued)*

Sr No	Variable	Description
91	selfambulatory__at__discharge	Was the patient able to ambulate on his own at discharge,
92	residents__confidence__in__managing__trauma__patients	

For peer review only

Supplemental Material 5

5.1 Interview: Patient Information Sheet

Patient Information Sheet

You are being invited to participate in a research study. Before you take part in this research study, the study must be explained to you and you must be given the chance to ask questions. Please read carefully the information provided here. If you agree to participate, please sign the informed consent form. You will be given a copy of this document to keep with you.

<u>Protocol Title: </u>Trauma Training Effectiveness Research Network

Principal Investigator: Name

PURPOSE OF THE RESEARCH STUDY

We are currently conducting research in this hospital to study the feasibility of assessing the effect of trauma life support training programs on care and outcome of patients with injury. Trauma means when a person has injuries that are serious. To select the outcomes most relevant to patients like you, we want to know what are the challenges you faced in returning back to normal life after the injury. We ask you to participate in this study because you presented to this hospital after having an injury.

STUDY PROCEDURES AND VISIT SCHEDULE

If you agree to participate, we will call you or a relative three months (90 days) after you arrived at this hospital to hear how you are. We will ask you for permission to visit you at your home. If you agree we will visit you at a time convenient to you to talk to you about health after discharge. You do not have to answer certain questions if you prefer not to. You can also end the interview whenever you want, even if all questions have not been answered. An audio recording of the interview may be taken.

WITHDRAWAL FROM STUDY

Participation in this study is completely voluntary. Even if you agree to participate now you are free to withdraw at any time without giving any reason for doing so. Withdrawing will not affect your ordinary treatment or the care given to you. To withdraw you contact any of the study contact persons on the numbers or emails listed below.

POSSIBLE RISKS, DISCOMFORTS AND INCONVENIENCES

We have not been able to identify any major risks associated with participating in this study. If you would at that point, or any other point of time, wish to withdraw from the study, you are free to do so.

POTENTIAL BENEFITS

Our research may help to study the most relevant outcomes for you to get back to normal life after the injury. Although this research will not affect the care you were given in this hospital at this time, its results might help you if you are injured again in the future, or

For peer review only

others that are injured. There is no assurance you will benefit from this study. However, your participation may contribute to the medical knowledge about the effect of the use of trauma life support training programs on the life of injured patients.

SUBJECT'S RIGHTS

Your participation in this study is entirely voluntary. Your questions will be answered clearly and to your satisfaction. In the event of any new information becoming available that may be relevant to your willingness to continue in this study, you will be informed in a timely manner by the Principal Investigator or his/her representative.

CONFIDENTIALITY OF STUDY AND MEDICAL RECORDS

The results of this research may be published as a scientific article; however, it will not be possible to identify you by reading any article that may result from this work. Further, data from this project will be shared with other researchers in India and abroad, but it will not be possible to identify you using only that data.

COSTS OF PARTICIPATION

If you take part in this study, there will be no charge levied on you. You will not receive any compensation for participating in this study.

RESEARCH RELATED INJURY AND COMPENSATION

The study being observational is not likely to cause any research related injury.

WHOM TO CONTACT IF YOU HAVE QUESTIONS

If you have questions about this research study and your rights or in the case of any injuries during the course of this study, you may contact:

Name
Department
Phone Number
Email

For peer review only

5.2 Interview: Patient Consent Form

Consent Form

Protocol Title: Trauma Training Effectiveness Research Network

Subject's Particulars

Name:

Address:

Date of birth ________________ Phone No

(dd/mm/yyyy)

I, __________________________________ **agree / do not agree** to participate in the research study as described and on the terms set out in the Patient Information Sheet. The nature of my participation in the proposed research study has been explained to me by Dr/Mr/Ms ______________________I have fully discussed and understood the purpose and procedures of this study. I have been given the Patient Information Sheet and the opportunity to ask questions about this study and have received satisfactory answers and information.

I understand that my participation is voluntary and that I am free to withdraw at any time, without giving any reasons and without my medical care being affected.

I also give permission for information in my medical records to be used for research. In any event of publication, I understand that this information will not bear my name or other identifiers and that due care will be taken to preserve the confidentiality of this information.

___________________________________ _________________________

[Signature/Thumbprint (Right / Left) of patient] (Date of signing)

For peer review only

Supplemental Material 6

6.1 Interview: Non-patient participant Information Sheet

Participant Information Sheet

You are being invited to participate in a research study. Before you take part in this research study, the study must be explained to you and you must be given the chance to ask questions. Please read carefully the information provided here. If you agree to participate, please sign the informed consent form. You will be given a copy of this document to keep with you.

<u>Protocol Title: </u>Trauma Training Effectiveness Research Network

Principal Investigator: Name

PURPOSE OF THE RESEARCH STUDY

We are currently conducting research in this hospital to study the feasibility of assessing the effect of trauma life support training programs on care and outcome of patients with injury. Trauma means when a person has injuries that are serious. To select the outcomes most relevant to patients we want to know what are the challenges patients face in returning back to normal life after the injury.

STUDY PROCEDURES

If you agree to participate, we will visit you at a time convenient to you to talk to you about challenges trauma patients face. You do not have to answer certain questions if you prefer not to. You can also end the interview whenever you want, even if all questions have not been answered. An audio recording of the interview may be taken.

WITHDRAWAL FROM STUDY

Participation in this study is completely voluntary. Even if you agree to participate now you are free to withdraw at any time without giving any reason for doing so. To withdraw you contact any of the study contact persons on the numbers or emails listed below.

POSSIBLE RISKS, DISCOMFORTS AND INCONVENIENCES

We have not been able to identify any major risks associated with participating in this study. If you would at that point, or any other point of time, wish to withdraw from the study, you are free to do so.

POTENTIAL BENEFITS

Our research may help to study the most relevant outcomes for patients to get back to normal life after the injury. There is no assurance you will benefit from this study. However, your participation may contribute to the medical knowledge about the effect of the use of trauma life support training programs on the life of injured patients.

PARTICIPANT'S RIGHTS

For peer review only

Your participation in this study is entirely voluntary. Your questions will be answered clearly and to your satisfaction. In the event of any new information becoming available that may be relevant to your willingness to continue in this study, you will be informed in a timely manner by the Principal Investigator or his/her representative.

CONFIDENTIALITY OF THE STUDY

The results of this research may be published as a scientific article; however, it will not be possible to identify you by reading any article that may result from this work. Further, data from this project will be shared with other researchers in India and abroad, but it will not be possible to identify you using only that data.

COSTS OF PARTICIPATION

If you take part in this study, there will be no charge levied on you. You will not receive any compensation for participating in this study.

RESEARCH RELATED INJURY AND COMPENSATION

The study being observational is not likely to cause any research related injury.

WHOM TO CONTACT IF YOU HAVE QUESTIONS

If you have questions about this research study and your rights during the course of this study, you may contact:

Name

Designation, Department

Phone Number

Email

For peer review only

6.2 Interview: Non-patient participant Consent Form

Consent Form

Protocol Title: Trauma Training Effectiveness Research Network

Subject's Particulars

Name:

Address:

Date of birth ________________ Phone No

(dd/mm/yyyy)

I, ___________________________ **agree / do not agree** to participate in the research study as described and on the terms set out in the Participant Information Sheet. The nature of my participation in the proposed research study has been explained to me by Dr/Mr/Ms ______________________I have fully discussed and understood the purpose and procedures of this study. I have been given the Participant Information Sheet and the opportunity to ask questions about this study and have received satisfactory answers and information.

I understand that my participation is voluntary and that I am free to withdraw at any time, without giving any reasons.

In any event of publication, I understand that it will not bear my name or other identifiers and that due care will be taken to preserve the confidentiality of this information.

____________________________________ ___________________________

[Signature/Thumbprint (Right / Left) of participant] (Date of signing)

For peer review only

Supplemental Material 7

Table S7.1: Shows which outcomes that will be assessed in which subgroups.

	All patients	Men	Women	Blunt multisystem	Penetrating	Shock	Severe traumatic brain injury	Elderly
All cause mortality within 30 days from the time of arrival to the emergency department	Yes	Yes	Yes	Yes	Yes	Yes	Yes	Yes
All cause mortality within 24 hours from the time of arrival to the emergency department	Yes	Yes	Yes	Yes	Yes	Yes	Yes	Yes
Time to all cause mortality during follow up.	Yes	Yes	Yes	Yes	Yes	Yes	Yes	Yes
Cause-specific in-hospital mortality.	Yes	Yes	Yes	Yes	Yes	Yes	Yes	Yes
Adherence to the WHO trauma care checklist.	Yes	Yes	Yes	Yes	Yes	Yes	Yes	Yes
Fluids for resuscitation in first one hour in patients.	Yes	No	No	No	No	Yes	No	No

For peer review only - http://bmjopen.bmj.com/site/about/guidelines.xhtml

Table S7.1: Shows which outcomes that will be assessed in which subgroups. *(continued)*

	All patients	Men	Women	Blunt multisystem	Penetrating	Shock	Severe traumatic brain injury	Elderly
Massive transfusion, defined as four or more units of packed red blood cells, plasma or platelets transfused within the first 24 hours after arrival to the emergency department.	Yes	No	No	No	No	Yes	No	No
Time to first surgery.	Yes	No	No	No	No	Yes	No	No
Time to first intubation.	Yes	No	No	No	No	No	No	No
Time to CT scan.	Yes	No	No	No	No	No	No	No
Ventilator free days.	Yes	No	No	No	No	No	No	No
ICU free days.	Yes	No	No	No	No	No	No	No
Pulmonary complications.	Yes	No	No	No	No	No	No	No
Septic shock.	Yes	No	No	No	No	No	No	No
Renal failure.	Yes	No	No	No	No	No	No	No
Coagulopathy.	Yes	No	No	No	No	No	No	No
Length of stay.	Yes	No	No	No	No	No	No	No
Quality of life.	Yes	No	No	No	No	No	No	No
Number of hospitalizations after the index admission during the follow up period.	Yes	No	No	No	No	No	No	No
Return to work.	Yes	Yes	Yes	Yes	Yes	Yes	Yes	No

For peer review only

Table S7.1: Shows which outcomes that will be assessed in which subgroups. *(continued)*

	All patients	Men	Women	Blunt multisystem	Penetrating	Shock	Severe traumatic brain injury	Elderly
Need for unplanned re-exploration.	Yes	No	No	No	No	No	No	No
Failure of non-operative management.	Yes	No	No	No	No	No	No	No
Patient satisfaction.	Yes	Yes	Yes	Yes	No	No	No	No
Out-of-pocked expenditure.	Yes	No	No	No	No	No	No	No
Self-ambulatory.	Yes	No	No	No	No	No	No	No

For peer review only

STANDARD PROTOCOL ITEMS: RECOMMENDATIONS FOR INTERVENTIONAL TRIALS

SPIRIT 2013 Checklist: Recommended items to address in a clinical trial protocol and related documents*

Section/item	Item No	Description	Addressed on page number
**Administrative information**			
Title	1	Descriptive title identifying the study design, population, interventions, and, if applicable, trial acronym	1
Trial registration	2a	Trial identifier and registry name. If not yet registered, name of intended registry	3
	2b	All items from the World Health Organization Trial Registration Data Set	Included
Protocol version	3	Date and version identifier	1
Funding	4	Sources and types of financial, material, and other support	1
Roles and responsibilities	5a	Names, affiliations, and roles of protocol contributors	1 - 3
	5b	Name and contact information for the trial sponsor	
	5c	Role of study sponsor and funders, if any, in study design; collection, management, analysis, and interpretation of data; writing of the report; and the decision to submit the report for publication, including whether they will have ultimate authority over any of these activities	1
	5d	Composition, roles, and responsibilities of the coordinating centre, steering committee, endpoint adjudication committee, data management team, and other individuals or groups overseeing the trial, if applicable (see Item 21a for data monitoring committee)	Not applicable

Introduction

Background and rationale	6a	Description of research question and justification for undertaking the trial, including summary of relevant studies (published and unpublished) examining benefits and harms for each intervention	6
	6b	Explanation for choice of comparators	6
Objectives	7	Specific objectives or hypotheses	6
Trial design	8	Description of trial design including type of trial (eg, parallel group, crossover, factorial, single group), allocation ratio, and framework (eg, superiority, equivalence, noninferiority, exploratory)	7

Methods: Participants, interventions, and outcomes

Study setting	9	Description of study settings (eg, community clinic, academic hospital) and list of countries where data will be collected. Reference to where list of study sites can be obtained	7
Eligibility criteria	10	Inclusion and exclusion criteria for participants. If applicable, eligibility criteria for study centres and individuals who will perform the interventions (eg, surgeons, psychotherapists)	7
Interventions	11a	Interventions for each group with sufficient detail to allow replication, including how and when they will be administered	8
	11b	Criteria for discontinuing or modifying allocated interventions for a given trial participant (eg, drug dose change in response to harms, participant request, or improving/worsening disease)	8
	11c	Strategies to improve adherence to intervention protocols, and any procedures for monitoring adherence (eg, drug tablet return, laboratory tests)	8
	11d	Relevant concomitant care and interventions that are permitted or prohibited during the trial	9
Outcomes	12	Primary, secondary, and other outcomes, including the specific measurement variable (eg, systolic blood pressure), analysis metric (eg, change from baseline, final value, time to event), method of aggregation (eg, median, proportion), and time point for each outcome. Explanation of the clinical relevance of chosen efficacy and harm outcomes is strongly recommended	9-10, 15-20
Participant timeline	13	Time schedule of enrolment, interventions (including any run-ins and washouts), assessments, and visits for participants. A schematic diagram is highly recommended (see Figure)	10

For peer review only - http://bmjopen.bmj.com/site/about/guidelines.xhtml

Sample size	14	Estimated number of participants needed to achieve study objectives and how it was determined, including clinical and statistical assumptions supporting any sample size calculations	10
Recruitment	15	Strategies for achieving adequate participant enrolment to reach target sample size	10
**Methods: Assignment of interventions (for controlled trials)**			
Allocation:			
Sequence generation	16a	Method of generating the allocation sequence (eg, computer-generated random numbers), and list of any factors for stratification. To reduce predictability of a random sequence, details of any planned restriction (eg, blocking) should be provided in a separate document that is unavailable to those who enrol participants or assign interventions	11
Allocation concealment mechanism	16b	Mechanism of implementing the allocation sequence (eg, central telephone; sequentially numbered, opaque, sealed envelopes), describing any steps to conceal the sequence until interventions are assigned	11
Implementation	16c	Who will generate the allocation sequence, who will enrol participants, and who will assign participants to interventions	11
Blinding (masking)	17a	Who will be blinded after assignment to interventions (eg, trial participants, care providers, outcome assessors, data analysts), and how	11
	17b	If blinded, circumstances under which unblinding is permissible, and procedure for revealing a participant's allocated intervention during the trial	11
**Methods: Data collection, management, and analysis**			
Data collection methods	18a	Plans for assessment and collection of outcome, baseline, and other trial data, including any related processes to promote data quality (eg, duplicate measurements, training of assessors) and a description of study instruments (eg, questionnaires, laboratory tests) along with their reliability and validity, if known. Reference to where data collection forms can be found, if not in the protocol	11-12
	18b	Plans to promote participant retention and complete follow-up, including list of any outcome data to be collected for participants who discontinue or deviate from intervention protocols	11-12

For peer review only - http://bmjopen.bmj.com/site/about/guidelines.xhtml

Data management	19	Plans for data entry, coding, security, and storage, including any related processes to promote data quality (eg, double data entry; range checks for data values). Reference to where details of data management procedures can be found, if not in the protocol	11 - 12
Statistical methods	20a	Statistical methods for analysing primary and secondary outcomes. Reference to where other details of the statistical analysis plan can be found, if not in the protocol	13 - 14
	20b	Methods for any additional analyses (eg, subgroup and adjusted analyses)	13 - 14
	20c	Definition of analysis population relating to protocol non-adherence (eg, as randomised analysis), and any statistical methods to handle missing data (eg, multiple imputation)	13 - 14
**Methods: Monitoring**			
Data monitoring	21a	Composition of data monitoring committee (DMC); summary of its role and reporting structure; statement of whether it is independent from the sponsor and competing interests; and reference to where further details about its charter can be found, if not in the protocol. Alternatively, an explanation of why a DMC is not needed	13
	21b	Description of any interim analyses and stopping guidelines, including who will have access to these interim results and make the final decision to terminate the trial	13
Harms	22	Plans for collecting, assessing, reporting, and managing solicited and spontaneously reported adverse events and other unintended effects of trial interventions or trial conduct	Not applicable
Auditing	23	Frequency and procedures for auditing trial conduct, if any, and whether the process will be independent from investigators and the sponsor	Not applicable
**Ethics and dissemination**			
Research ethics approval	24	Plans for seeking research ethics committee/institutional review board (REC/IRB) approval	14
Protocol amendments	25	Plans for communicating important protocol modifications (eg, changes to eligibility criteria, outcomes, analyses) to relevant parties (eg, investigators, REC/IRBs, trial participants, trial registries, journals, regulators)	14

Consent or assent	26a	Who will obtain informed consent or assent from potential trial participants or authorised surrogates, and how (see Item 32)	12
	26b	Additional consent provisions for collection and use of participant data and biological specimens in ancillary studies, if applicable	Not applicable
Confidentiality	27	How personal information about potential and enrolled participants will be collected, shared, and maintained in order to protect confidentiality before, during, and after the trial	13
Declaration of interests	28	Financial and other competing interests for principal investigators for the overall trial and each study site	14
Access to data	29	Statement of who will have access to the final trial dataset, and disclosure of contractual agreements that limit such access for investigators	14
Ancillary and post-trial care	30	Provisions, if any, for ancillary and post-trial care, and for compensation to those who suffer harm from trial participation	Not applicable
Dissemination policy	31a	Plans for investigators and sponsor to communicate trial results to participants, healthcare professionals, the public, and other relevant groups (eg, via publication, reporting in results databases, or other data sharing arrangements), including any publication restrictions	14
	31b	Authorship eligibility guidelines and any intended use of professional writers	14
	31c	Plans, if any, for granting public access to the full protocol, participant-level dataset, and statistical code	14
**Appendices**			
Informed consent materials	32	Model consent form and other related documentation given to participants and authorised surrogates	Supplement 4-7
Biological specimens	33	Plans for collection, laboratory evaluation, and storage of biological specimens for genetic or molecular analysis in the current trial and for future use in ancillary studies, if applicable	Not applicable

*It is strongly recommended that this checklist be read in conjunction with the SPIRIT 2013 Explanation & Elaboration for important clarification on the items. Amendments to the protocol should be tracked and dated. The SPIRIT checklist is copyrighted by the SPIRIT Group under the Creative Commons "Attribution-NonCommercial-NoDerivs 3.0 Unported" license.
